# Homogeneous catalyst graph neural network: A human-interpretable graph neural network tool for ligand optimization in asymmetric catalysis

**DOI:** 10.1016/j.isci.2025.111881

**Published:** 2025-01-23

**Authors:** Eduardo Aguilar-Bejarano, Ender Özcan, Raja K. Rit, Hongyi Li, Hon Wai Lam, Jonathan C. Moore, Simon Woodward, Grazziela Figueredo

**Affiliations:** 1 GSK Carbon Neutral Laboratories for Sustainable Chemistry, University of Nottingham, Jubilee Campus, Triumph Road, Nottingham NG7 2TU, UK; 2 School of Chemistry, University of Nottingham, University Park, Nottingham NG7 2RD, UK; 3 School of Computer Science, University of Nottingham, Jubilee Campus, Wollaton Road, Nottingham, Nottingham NG8 1BB, UK; 4 School of Medicine, University of Nottingham, Medical School, Nottingham NG7 2UH, UK

**Keywords:** Artificial intelligence, Catalysis, Chemistry

## Abstract

Optimization of metal-ligand asymmetric catalysts is usually done by empirical trials, where the ligand is arbitrarily modified, and the new catalyst is re-evaluated in the lab. This procedure is not efficient and alternative strategies are highly desirable. We propose the Homogeneous Catalyst Graph Neural Network (HCat-GNet), a machine learning model capable of aiding ligand optimization. This method trains models to predict the enantioselectivity of asymmetric reactions using only the SMILES representations of the participant molecules. HCat-GNet allows high interpretability indicating from which atoms the model gathers the most predictive information, thus showing which atoms within the ligand most affect the increase or decrease in the reaction’s selectivity. The validation of the model’s selectivity predictions is made using a new class of ligand for rhodium-catalyzed asymmetric 1,4-addition, demonstrating the ability of HCat-GNet to extrapolate into unknown chiral ligand space. Validation with other benchmark asymmetric reaction datasets demonstrates its generality when modeling different reactions.

## Highlights

• Enantioselectivities predictions of any metal-chiral ligand catalyzed reaction
• Good performance using only SMILES representations
• Simple general methodology using only benchtop computing
• Explainable AI allows human understanding of ligand (L∗) motifs affecting the selectivity

## Graphical abstract

**Figure:**
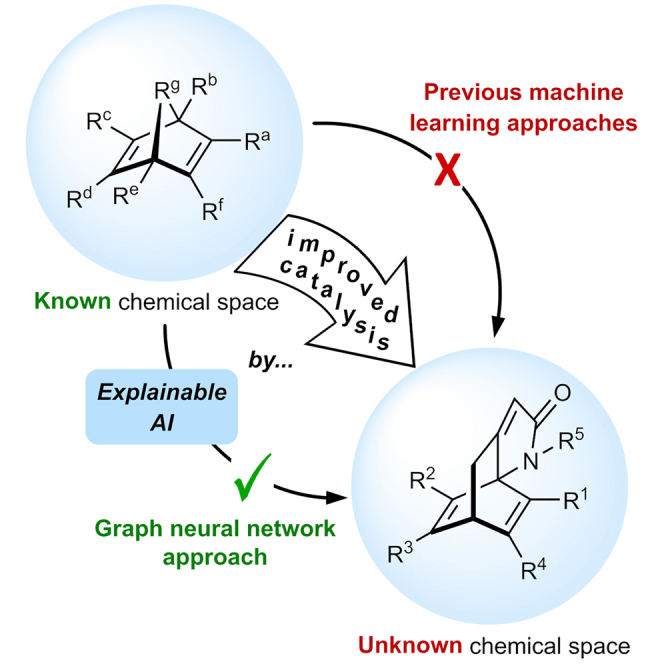

## Introduction

**Figure 1:**
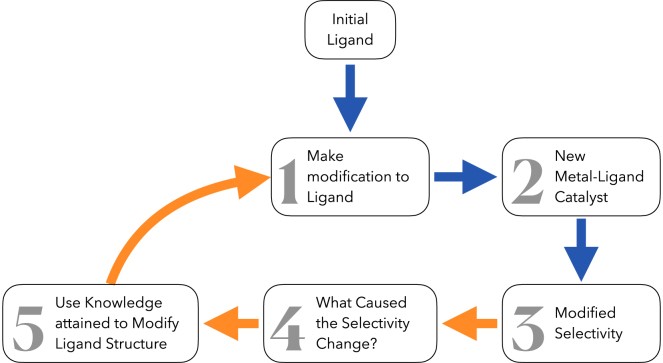
Typical workflow for ligand optimization for asymmetric catalysis

The structural optimization of the chiral ligand (L∗) used in a new metal-ligand (ML∗)-catalyzed asymmetric reaction is frequently time-demanding. Typical workflows (Figure 1) involve the chemical modification of the ligand (L∗), formation of new complexes (ML∗), testing of a benchmark reaction to reveal its experimental enantioselectivity (either *ee* or *er*), human rationalization of the steric, electronic or other factors that might be responsible for the selectivity change and finally the formation of new derivatives to confirm (or deny) those hypotheses. Frequently, only small numbers of new ligand structure-selectivity relationships are available in the early stages of such workflows, as each new L∗ can (sometimes) take days (or more) to prepare and assess.

Machine learning has gained popularity in chemical science as it allows quantitative structure-activity relationship (QSAR) studies in efficient, computationally inexpensive yet accurate ways.^1^^,^^2^^,^^3^ Typically, *ad hoc* descriptors that model the chemical structure are used to represent the molecule computationally.^4^ However, Dos Passos Gomes et al. highlighted the paucity of machine learning approaches in asymmetric catalysis and the need for more research in this area.^5^ Similarly, Hirst et al. and Kalikadien et al. encourage the use of techniques to aid ML∗ catalyst discovery.^6^^,^^7^

Applications of machine learning for homogeneous ligated catalysts are known.^8^^,^^9^^,^^10^^,^^11^^,^^12^^,^^13^ However, just a handful of those target asymmetric catalysis by ML∗ catalysts.^14^^,^^15^^,^^16^^,^^17^^,^^18^ In one example, Bretholomé et al.,^19^ studied the enantioselectivity of nucleophiles to a Michael acceptor using a chiral PR^1^R^2^R^3^ ligand. Keeping two (R^1^,R^2^) substituents fixed, a linear regression between the reaction enantioselectivity and the *logP* of substituent R^3^ was used as a model to predict the enantioselectivity of *in silico* designed ligands. As only one ligand substituent (R^3^) is modeled, the time saved in the synthetic effort through the algorithm approach was limited.

**Figure 2:**
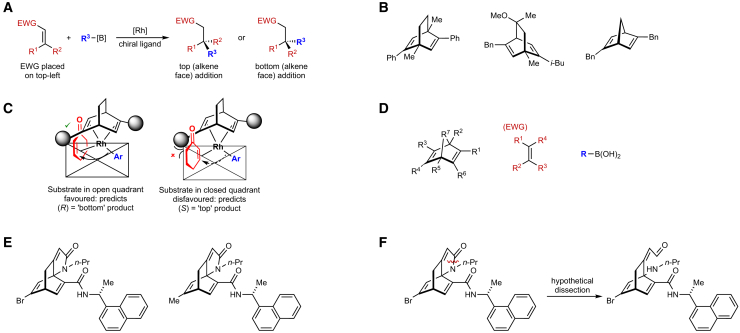
The RhCAA reaction and representative chiral ligands (A) General RhCAA, where the structure of the chiral ligand (L∗) determines the absolute stereochemistry of the product. (B) Representative chiral 1,4-diene ligands (L∗). (C) The steric selectivity model proposed by Hayashi (human expert).^21^ (D) Substituent representation used by Owen et al. in their descriptor generation procedure.^20^ (E) Examples of the new ligands developed by Rit et al. to improve pre-existing ligands.^45^ (F) The limitation of Owen’s approach: the new Rit ligands^45^ have to be “cut” (arbitrarily and hypothetically) to generate Owen’s descriptors.^20^

Owen et al. developed a general descriptor generation procedure for rhodium-catalyzed asymmetric 1,4-additions (RhCAA) (Figure 2A) catalyzed by chiral 1,4-diene ligands (Figure 2B).^20^ Inspired by an empirical selectivity model (Figure 2C),^21^ steric and electronic descriptors of substituents in the core structure of the substrate, chiral diene ligand, and organoboron reagent were used (Figure 2D). Owen et al. obtained satisfactory results with a range of algorithms when predicting reaction enantioselectivity (mean error ±10% *ee*). This simple approach is useful but requires lengthy human curation of the chiral diene ligand descriptors, which include (not always available for all possible substituents) Hammett parameters. In another study, Tsuji et al. proposed the use of circular substructures (CircuS) in catalysis,^22^ while Sandfort et al. proposed multiple fingerprint features for reaction performance prediction.^17^ They are a rare example of a reaction-agnostic approach that does not require time-intensive computation, but their scope and limitations have not yet been fully delineated.

Alternative approaches for predicting the enantioselectivity provided by ML∗ catalysts in asymmetric reactions are known.^23^^,^^24^^,^^25^ However, these methods have common shortcomings for use in the onward L∗ design required in Figure 1, specifically.

(1) Most methods are applied to a limited chemical space, where the training data nearly identical to the test data, restricting the approach’s ability to predict/explore new ligand (L∗) space.^20^^,^^23^^,^^24^^,^^25^
(2) Sometimes the algorithm is only able to optimize one variable at a time, e.g., a single ligand substituent.^19^^,^^23^
(3) Methods often lack human interpretability, which is vital to enable synthetic lab chemists to profit from the AI predictions by implementing structure-activity relationships.^9^^,^^14^^,^^15^^,^^26^

Considering these points, it is desirable to develop a reaction-agnostic, easy-to-use, interpretable method that allows fast prediction of the stereochemical outcomes for chiral ligated catalysts, which can be applied to a wider range of reactions and chemical space without significant computational (e.g., DFT) costs.

Graph neural networks (GNNs) are powerful tools for attaining quantitative structure-activity relationships (QSARs).^27^^,^^28^^,^^29^^,^^30^^,^^31^ GNN algorithms can discover high-level descriptors within molecular graphical representations of data allowing chemical predictions.^32^^,^^33^^,^^34^^,^^35^^,^^36^^,^^37^ Here we test the hypothesis that Graph Neural Networks (GNNs) derived directly only from pre-existing chiral ligand (L∗) structures are able to provide explainable insights comparable to human expert deductions into future ligand optimization (the orange arrow steps of Figure 1).

GNNs have commonly been used to accurately predict reaction yield^38^ and regioselectivities.^39^ In a single study, Hong and collaborators used GNNs for excellent stereoselectivity and yield predictions for an organo-catalytic enantioselective reaction.^40^ They used a knowledge-based graph that embedded DFT-derived steric and electronic information in combination with a molecular interaction module in a GNN architecture for their predictions. While his study shows the power of GNNs in modeling of asymmetric catalysis Hong’s feature generation relies on DFT calculations. Issues might arise: (i) accessibility to non-computational, laboratory-based communities and (ii) even semi-empirical DFT calculations can become computationally demanding when screening large libraries of *in silico* generated ligand targets, especially if metals are present.

**Figure 3:**
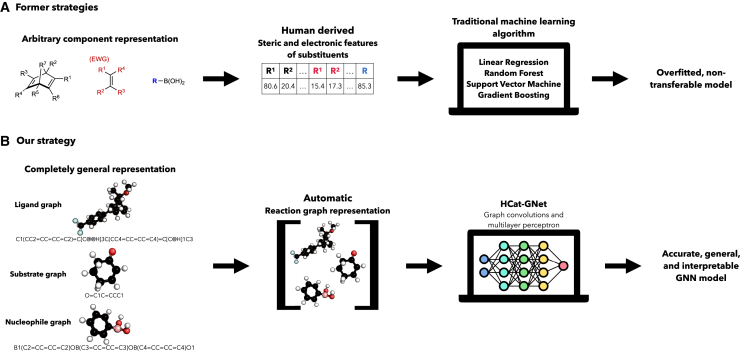
Strategies for the enantioselectivity prediction of RhCAA reactions (A) Traditional, human intervention featurization (slower) by Owen et al.^20^ (B) the automated HCat-GNet strategy described herein (faster).

As an alternative we have developed the homogeneous catalyst graph network (HCat-GNet), an interpretable GNN that can predict both the absolute stereochemistry of the product (*R* vs. *S*) and the selectivity expressed as a ΔΔG^‡^ value for asymmetric reactions driven by *any* chiral ML∗ species given *only* SMILES representations of the molecules involved in the transformation.^41^ We foresee two main advantages to this approach: (i) current machine learning systems infrequently deliver outputs that are readily interpretable by human chemists (e.g., that the substituent at a certain position should be small, aromatic and electron-rich), thus directly supporting human expert lab expertise. (ii) No recourse to (potentially) computationally expensive DFT modeling is required. To demonstrate its utility, HCat-GNet is used here for ligand (L∗) development predictions in rhodium-catalyzed asymmetric 1,4-additions (RhCAA) of organoboron reagents to prochiral Michael acceptors (Figure 2A).^21^^,^^42^^,^^43^^,^^44^ This is a useful as a human-expert ligand development “test set” cycle akin to Figure 1 has recently become available.^45^ We also compare the HCat-GNet approach to standard machine learning methods (as exemplified by Owen et al.^20^), using three different established machine learning algorithms (see Figures 2D and 3A). RhCAA is selected as our case study because: (i) many laboratory examples are available from the literature,^44^ leading to a richer dataset; (ii) pre-existing machine learning approaches are available as benchmarks^20^; and (iii) a historical simple steric stereochemical model for the process exists (Figure 2C),^21^ allowing comparison of our GNN approach against a human-derived explanation. We further benchmark HCat-GNet using other three datasets: a RhCASA reaction catalyzed by chiral diphosphine ligands dataset,^44^ a catalyzed asymmetric dearomatization using hypervalent iodine (III),^16^ and asymmetric *N*, *S*-acetal formation reaction dataset.^26^ We aimed to answer three questions: (i) Can GNNs accurately predict the enantioselectivity of asymmetric reactions in chiral ligand-based catalysis using simple graphical representations? (ii) How do GNN models perform in predicting the enantioselectivity of new development chiral ligands (structurally distinct from the training data and unseen by the algorithm)? (iii) Can GNN explainability algorithms be used to predict fragments or functional groups that increase/decrease the stereoselectivity of the reaction? If achieved, these goals would simplify the task of Figure 1.

## Results

### Homogeneous catalyst graph neural network graph representation

HCat-GNet is able to encode *a wide range of* asymmetric catalytic reactions with *any* number of reacting molecules and any ligand(s). We use a graph representation of each molecule component (e.g., ligand, substrate, reagent, and so forth) within the chiral reaction and a reaction-level (containing all react components) graph representation to predict reaction enantioselectivity.

In generating molecular representations, the algorithm iterates over all the atoms within a component molecule. For each atom (node), the algorithm retains information on it and another atoms sharing a bond with it, its atomic properties, including its identity, degree (number of non-hydrogen atoms are attached to it), hybridization, whether or not it is part of an aromatic system, whether or not it is part of a ring, and the absolute configuration of the stereocenter at that atom (either *R*, *S* or none). Component connectivity information is transcribed into an adjacency list, and the atomic property information is retained in a node feature matrix, one-hot-encoded. The adjacency list along with the node feature matrix is the graph representation of the molecule (see Figure S1). This pre-processing is conducted automatically for all molecule components within the reaction dataset.

Once all graphs have been generated, the molecular graphical representations are concatenated into a single disconnected graph, so that the resultant reaction graph consists of *n* sub-graphs (where *n* is the number of participant molecules) (see Figure S2). The node feature matrix is concatenated as well, and this along with the adjacency list, is the complete reaction-graph representation. This approach is agnostic to the reaction for *all* ML∗-based catalytic reactions, as it can accommodate any number of reaction components or ligands without algorithmic modification.

For RhCAA in our datasets, there are three key molecular components: the chiral ligand, substrate, and organoboron reagent, which are automatically converted into molecular graphs from just their SMILES strings. Initially, the HCat-GNet takes the SMILES representation of the ligand and creates a graph representation (see above), then does the same for the substrate and lastly for the organoboron reagent. We add a graph-level feature to the ligand graph, called “configuration” that encodes the planar chirality of the ligand without recourse to time-consuming (manual), stereochemical assignment of all planar chirality ligand elements^21^ (Figures S1-S3 related to STAR Methods RhCASA ‘seen’ curated dataset). As the selectivity of asymmetric catalytic reactions is strongly affected by temperature, we include this as a system-level descriptor shared by all three graphs (see Figure S1). We do not use the edge features, as node level representation sufficiently represents all steric and electronic effects. Lastly, the algorithm creates the reaction-graph representation by concatenating the three molecular graph representations (see Figure 3B).

### Homogeneous catalyst graph neural network architecture

HCat-GNet has two phases: message passing and readout. The first phase is a node-level operation block, which explores the topology of the graph to capture the complex relations between neighboring nodes. This operation is known as convolution. We have used the Graph Convolutional Operator,^46^ inspired from the usual convolutional operator applied to images, which can also be generalized to the graph structures where each node can have different numbers of neighbors. This operator is defined as shown in Equation 1, where *i* is the central node that needs to be updated, $h_{i}^{\left(l\right)}$ is the node *i* current state, j∈N(i)∪{i} represents all the neighbors *j* of *i* (including *i* itself), ${\hat{d}}_{x}$ represents the number of neighbors of node *x*, $W^{\left(l\right)}$ is a learnable matrix, and $h_{i}^{\left(l+1\right)}$ is the new state of the central node *i*. This process is run in parallel for all nodes within the system so that each node receives new node features after each convolution, which can be run as many times as desired. Importantly, as the participant molecules are represented as separated graphs, the convolution operation does not allow information sharing between nodes of different molecules.

(Equation 1) $$h^{\left(l+1\right)}=\sum_{j\in N\left(i\right)\cup \left\{i\right\}}\frac{1}{\sqrt{\hat{d_{i}}\hat{d_{j}}}}h_{j}^{\left(l\right)}W^{\left(l\right)}$$

The second phase is the predictive task at the graph level. Initially, information from all the nodes contained within each graph is summarized into a single graph-level feature vector by an operation called pooling. This operation is an element-wise operation that runs for all the node feature vectors contained in the graph representation. Vitally, this allows encoding all molecules within the database, even those with different numbers of atoms (e.g., ligand, substrate, and organoboron reagent in RhCAA). Pooling ensures that the different sized molecules represented by graphs (different numbers of nodes) converge to a single fixed size vector regardless of the initial number of atoms (nodes) in the reaction (substrate, organoboron reagent, and ligand). The latter vector serves as an input into a multilayer perceptron (MLP) that outputs a final prediction. For HCat-GNet the steps from graph input to prediction are.

(1) The node features are taken (25 in total for all atoms in all molecules, see Figure S1) and these are expanded to a final length of 64 by a graph convolutional operator with leaky ReLU activation function, R^nodes×25^ → R^nodes×64^.
(2) The graph convolution operator updates all the node states in parallel, updating the node features to graph aware features once with leaky ReLU activation function, R^nodes×64^ → R^nodes×64^.
(3) Mean and max pooling is applied elementwise to all the node feature vectors to get a graph-level feature vector, R^nodes×64^ → R^1×128^.
(4) A fully connected layer with a leaky ReLU activation function takes the graph-level vector and maps it to half of its length, R^1×128^ → R^1×64^.
(5) A last fully connected layer transforms the feature vector into a scalar number, this being the prediction of the model, R^1×64^ → R^1^.

### Interpretability of homogeneous catalyst graph neural network

Explainable Artificial Intelligence (XAI) can be applied to HCat-GNet to provide significant insights into those chemical patterns that contribute the most in making the chiral catalyst (ligand, L∗) deliver higher or lower enantioselectivity. We use GNNExplainer,^47^ implemented in PyTorch Geometric 2.4.0, to understand the relevance of each node feature within the graph representation of the reaction, and Shapley Value Sampling^48^^,^^49^ (also implemented in PyTorch) to understand the contribution of each node (atom) within the graph to the enantioselectivity of the reaction.

GNNExplainer identifies subgraphs and subsets of the node features that are more influential for the model’s prediction^47^ using Equation 2. Here MI quantifies the change in the probability of prediction $\hat{y}=\Theta \left(G_{c},X_{c}\right)$ , where *G*_*c*_ is the complete graph and *X*_*c*_ are the complete node features, when the computation graph is limited to the subgraph *G*_*s*_ and node features limited to *X*_*s*_, and *H* is the entropy (uncertainty) of the model’s prediction *Y*. Effectively, GNNExplainer aims to remove edges, nodes, and node features that add to the uncertainty of the prediction. We use this algorithm to explore which node-level properties mostly drive the model to predict stereoselective outcomes for each molecule separately (ligand, substrate, and organoboron reagent).

(Equation 2) $$\max\;MI\left(Y,\left(G_{s},X_{s}\right)\right)=H\left(Y\right)-H\left(Y|G_{s},X_{s}\right)$$

Shapley Value Sampling (SHAP) is a perturbation method that computes an attribution score for descriptive node features within a graph using cooperative game theory.^50^ SHAP takes random permutations of the input graph node features and relates these to the real values of the feature baselines. The output gives the feature’s attribution (importance).^48^^,^^49^ SHAP provides an understanding of positive or negative node feature impact on the GNN prediction GNN allowing the most important fragments within the reacting molecules to be identified.

### Rhodium-catalyzed asymmetric 1,4-addition case study

#### Datasets

To test the efficacy of HCat-GNet as a tool for aiding the design of new chiral ligands we took a library of 668 reactions catalyzed by known diene ligands historically used for RhCAA^20^^,^^44^ (see Methods S1: data augmentation strategy and Figures S4, S6 and S14 relating to Figures 2A–2D), which we refer to as the “seen” set. Firstly, we confirmed that the GNN models these data, as well as other common machine learning algorithms. Secondly, we tested the effectiveness of the trained models in predicting the enantioselectivity of chiral diene ligands from a *real experimental* (human) optimized ligands^45^ (structures in Figures 2D and 2E, with the distribution of Figure S5, from the data of Tables S3 and S4), which we refer as the “unseen” set (see Methods S2: Preparation and use of “unseen” ligands, related to Figure 2). Thirdly, we extract which steric, electronic, or other factors drive the selectivity change in the new (unseen in training) ligands. The latter two tests simulate the use of HCat-GNet as a ligand development tool in minimizing the number of new ligands that must be synthesized in the lab to optimize the new asymmetric process (Figure 1). The “seen” set contains a total of 150 different chiral ligands, 84 substrates, and 45 boron reagents. The “unseen” set contains a total of 36 ligands (none of them in the “seen” set), 6 substrates (3 of those included in the “seen” set), and 3 boron reagents (the 3 included in the “seen” set).

#### Data labeling

Stereocenter assignment by the Cahn-Ingold-Prelog (CIP) *R/S* approach depends on the identity of the R^1^/R^2^ groups within the substrate (Figure 2A). It is possible for two reactions with the same innate facial selectivity (i.e., the “top” as drawn in Figure 2A) to show the opposite CIP assignment on changing the R^1^/R^2^ group identity. This makes the prediction task for the machine harder, as it has to learn both the facial selectivity of the reaction and the CIP rules. Thus, we use the “%top” variable adopted by Owen et al.,^20^ which consists of the percentage of the addition of the nucleophile to the “top” face of the substrate, as defined by the face of the substrate seen when placing the electron-withdrawing group (EWG) on the top left corner of the alkene (Figure 2A).^20^ The selectivity from the databases (originally enantiomeric excess and enantiomeric ratio) was recalculated in terms of %top. From this variable, we attain ΔΔG^‡^ (in kJ mol^−1^, STAR Methods: ΔΔG‡ target variable calculation). The value ΔΔG^‡^ is the difference in energy between the two key transition states providing the different stereochemical outcomes (“top” and “bottom”). Positive values of ΔΔG^‡^ denote that the major product is the “top” product, while negative values denote the production of excess “bottom” product. By relabeling the data, the models learn both the selectivity of the process (given by the numerical value of the prediction of ΔΔG^‡^) and the facial selectivity of the reaction (given by the sign of the prediction of ΔΔG^‡^). The latter is useful in comparing the general stereoselectivity of a range of ligands.

#### Prediction task

We predict two variables either the face of addition (“top” or “bottom”) or ΔΔG^‡^. The former is a classification problem, where “1” represents those reactions where the major product is the “top” isomer (%top >50%, or ΔΔG^‡^ > 0), and “0” otherwise. To convert the continuous regression predictions of the machine learning models into discrete values, predicted values greater than 50% are considered as predictions of the “top” isomer, and “bottom” otherwise. For the ΔΔG^‡^ regression task, the predicted values are those delivered by the algorithm.

#### Comparison to benchmark descriptors

We used Owen’s descriptors to train non-GNN algorithms to compare the effectiveness of HCat-GNet.^20^ Electronic and steric substituent descriptors as a function of their different positions within the ligands, substrates, and organoboron reagents were used (these positions are shown Figures 2D and 3A).^20^^,^^51^^,^^52^ In Owen’s approach, each of the substituents of Figure 2D are given descriptors of their estimated van der Waals Volume using Zhao’s method,^52^ and their electronic contribution by their Hammett parameter taken from an extensive review.^51^ This molecule encoding process leads to a total of 19 descriptors, which must be laboriously curated manually. To allow ΔΔG^‡^ prediction we also added a temperature descriptor. For the “unseen” dataset (Rit ligands,^45^ see Tables S3 and S4 related to Figure 2E), their featurization required an adaption of Owen’s^20^ approach. In the Rit ligands, the R^2^ and R^7^ substituents are connected through a lactam, and an arbitrary “cut” must be made to allow the procedure of Owen (Figure 2D) to be applied. Human chemical expertise suggested the virtual “cut” shown in Figure 2F was the most appropriate, but clearly others could have been made (with potentially different data outcomes). This is a potential limitation of the approach of Owen.^20^ We also used the CircuS representation as an additional benchmark.^22^ We chose this due to its ability to make enantioselectivity predictions by a reaction-agnostic workflow that also do not require DFT calculation. We found this benchmark to be less competitive than the approach of Owen^20^ and HCat-GNet with “unseen” data (Figure S12).

#### Model training and evaluation

To evaluate the transferability and robustness of all methods, we used a stratified nested cross-validation approach, as developed by García et al.^53^ (STAR Methods supported by Figure S7) for the “seen” dataset. We created 10-folds, which led to ten test sets, each evaluated by nine training and validation sets (each set consisting of 67 or 66 reactions). This increased to a total of 90 training-testing processes. The folds used in the Owen’s machine learning approach^20^ and HCat-GNet are identical, meaning that both methods are trained and evaluated using the same set of points. For Owen’s approach,^20^ Linear Regression, Random Forest, and Gradient Boosting were used as machine learning algorithms. For the face of the addition task, we evaluate the models using the metrics of Precision, Recall, and Accuracy. In the case of the ΔΔG^‡^ task, we report the metrics of mean absolute error (MAE), root mean squared error (RMSE), and determination coefficient (R^2^). We show the mean of the metrics for each test set separately, and standard deviation as error bars. This means that although we treat the classical diene set as a “seen set,” within this set, a representative sub-set of the data is used to deliver an unbiased evaluation of all models, whose results we report in this article (metrics of training and validation sets are available in the STAR Methods: key resources table). For the case of prediction selectivity of the 52 “unseen” dataset reactions (compromising the completely “unseen” new ligands), we took the 90 models trained with the “seen” dataset, with no further training. Predicting the enantioselectivity this way models the AI-led ligand design process foreseen for Figure 1.

### Homogeneous catalyst graph neural network performance

We evaluate HCat-GNet against traditional machine learning methods using different representations (bespoke by Owen^20^ and CircuS^22^). We noticed that bespoke descriptors performed better than the CircuS, and thus, we present the results of CircuS only in Supplemental Information (see Figure S12) and compare HCat-GNet against the best performing bespoke descriptor (Owen^20^) approach here. We compare these results for both “seen” (historical ligands^44^) and “unseen” (new Rit-ligand candidates^45^) datasets. Results of additional non-GNN algorithm benchmarks are also available (see Figures S8 and S9).

### Homogeneous catalyst graph neural network applicability to homogeneous catalysis analysis

**Figure 4:**
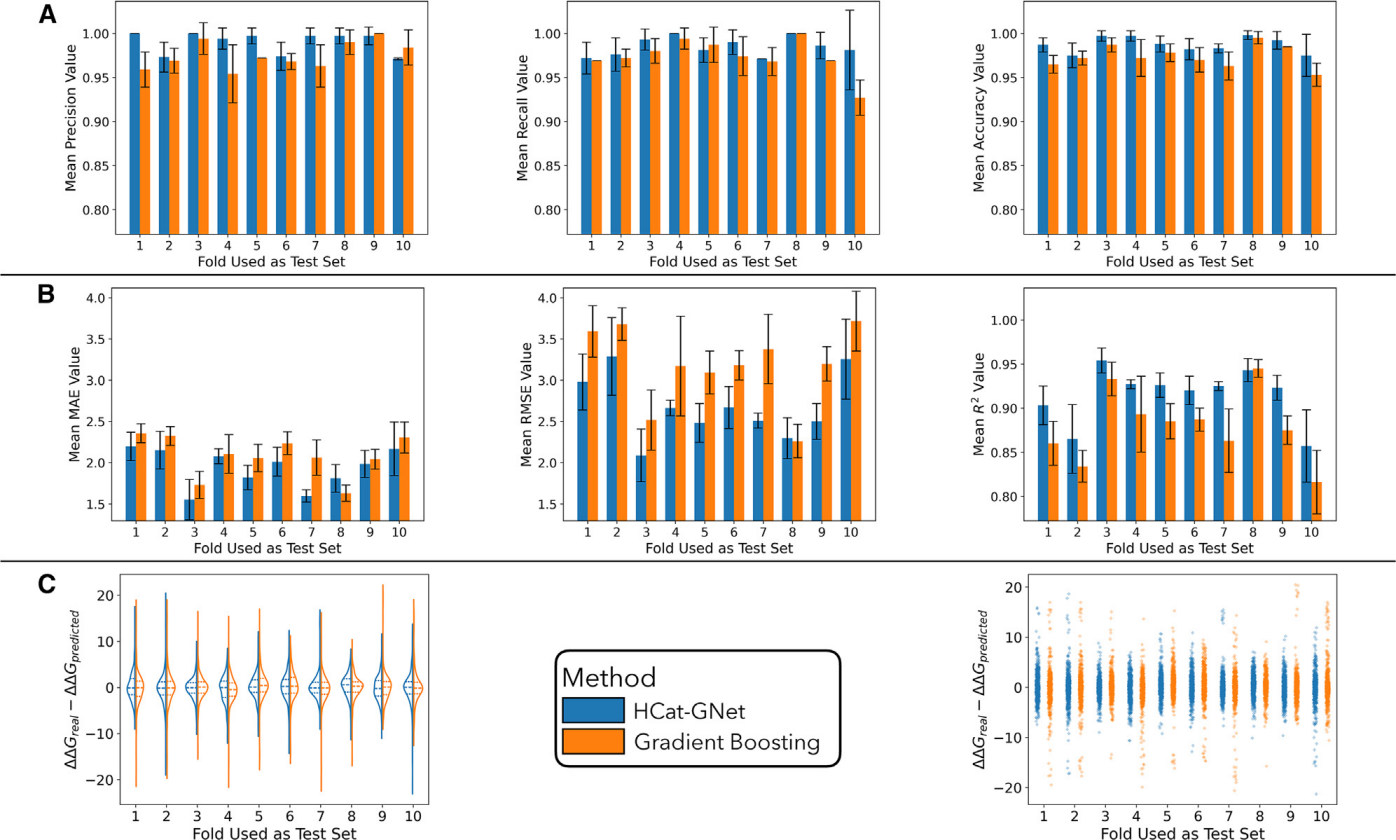
Benchmarking of HCat-GNet () against Gradient Boosting (, the best performing competitor of all tried benchmark alternatives) for the “seen” dataset Values are shown for each test fold separately, as the mean of the metrics for the value and the standard deviation as error bars. (A) Prediction of enantioface of addition (classification task), (B) prediction of enantioselectivity (ΔΔG^‡^ regression task), and (C) error distribution of all the predicted points for all train-testing folds. The color key in (C) applies to all the metrics shown. Data are represented as mean metrics, and error bars show the STD of each mean.

In Figure 4A we show the results for predicting the enantioface of the addition of the reagent to the substrate, comparing the GNN and Gradient Boosting methods. From the classification metrics, it is noticeable that both models accurately predict the facial selectivity of the reaction given the molecular representation of the participant molecules. In the case of the Gradient Boosting, this is entirely due to the encoding used by Owen et al.^20^ as fixed substituent enumeration rules for R^1^-R^7^ (Figure 2D) on the chiral diene implicitly encode the chirality of the ligand (L∗). The high accuracy of HCat-GNet for enantioface prediction can be attributed to the overall configuration feature added to the chiral ligand graph.

**Figure 5:**
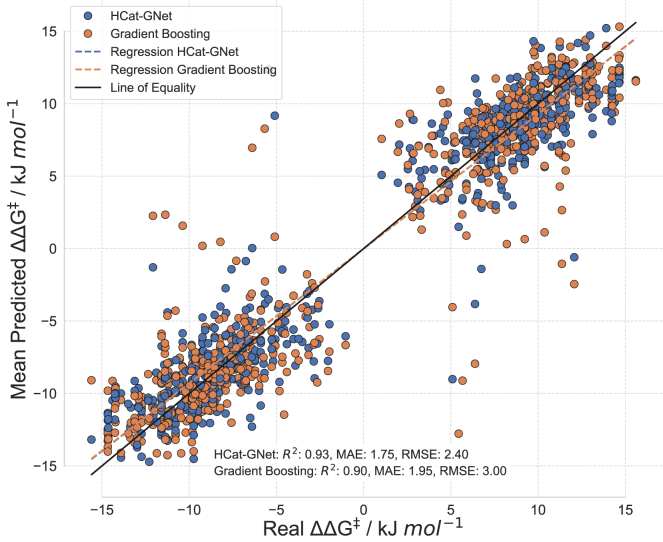
Parity plot of mean predicted ΔΔG^‡^ from the nine different training processes for each test point against the experimental ΔΔG^‡^, benchmarking HCat-GNet () against Gradient Boosting with the best bespoke (Owen^20^) competitor features () for the “seen” dataset The Wilcoxon test *p*-value of 0.825 indicates no overall statistical difference between these predictions. Mean predicted values and standard deviations are available (Data S1).

The regression task metrics (Figure 4B) indicate both algorithms provide satisfactory results, with mean absolute errors of 2.5 kJ mol^−1^ or less on the predicted selectivity. The error obtained by the models is so small that it can be attributed to experimental variance or error in the real-world data (in terms of enantiomeric ratio for a reaction that exhibits 10 kJ mol^−1^ in selectivity, the error is lower than 5% *er*). The RMSE of Gradient Boosting has larger values and has larger variance indicating this algorithm is more susceptible to outliers even though both methods show similar overall performance. The violin plot in Figure 4C shows both methods generate similar error distributions. However, from the strip plot, it is seen that the Gradient Boosting potentially leads to a larger quantity of significant errors (up to 20 kJ mol^−1^). To analyze the performance of both models in greater depth, we created a parity plot of the mean predicted ΔΔG^‡^ values from the 9 different testing processes applied to each test fold and the experimental ΔΔG^‡^, shown in Figure 5.

A parity plot (Figure 5) confirms both methods generate predictions with high accuracy but that Gradient Boosting has more outliers. By manual inspection, we identified a few reactions that were predicted with absolute errors ≥5 kJ mol^−1^ for both algorithms. There was little commonality between the two error sets, suggesting that the Gradient Boosting and HCat-GNet methods learned different correlations between the reaction representations used and the derived enantioselectivity outcomes. HCat-GNet and Gradient Boosting can both be used for ligand ranking within the “seen” dataset. However, HCat-GNet has the advantage of being trained automatically from the SMILES representation of molecules. For the Gradient Boosting approach of Owen et al.^20^ it is necessary to manually find and input van der Waals volumes and Hammett parameters (which must be found from an appropriate review) which is time-consuming.

### Model transferability evaluation

**Figure 6:**
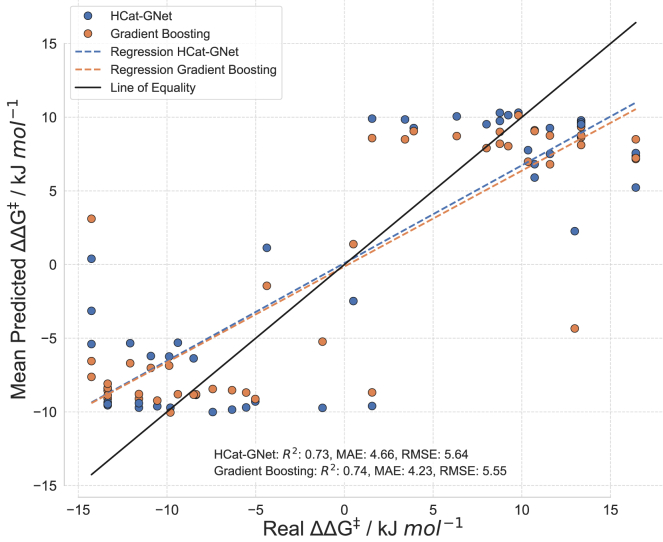
Testing HCat-GNet’s () ability to rank “unseen” ligand development candidates (by ΔΔG^‡^ comparison) when hunting for new improved chiral ligands against Gradient Boosting () The parity plot keeps the “unseen” dataset^45^ outside of the nested cross-validation approach. A Wilcoxon test shows a *p*-value of <0.01 indicating the statistical difference between the predictions of these methods. Mean predicted values and standard deviations are available (Data S1).

Our main goal for HCat-GNet is its use to aid chiral ligand (L∗) optimization, especially at early stages where new lab-based experimental ligand data is sparse (Figure 1). Recently, our group reported the synthesis of chiral Himbert diene ligands (Figure 2E), which show high enantioselectivity in RhCAA for a wide range of different substrates.^45^ We attained this position empirically, but the family of human-designed development ligands (see Methods S2: preparation and use of “unseen” ligands, relates to Figure 2) does allow us to explicitly test the utility of HCat-GNet as an L∗ design co-pilot. By not allowing the GNN algorithm to train further on these truly “unseen” data we can determine if HCat-GNet can perform as well as a human expert and compare its performance if we subsequently add some of this new “unseen” dataset to its training. Figure 6 shows the parity plots set off these two test scenarios.

Both HCat-GNet and Gradient Boosting as a benchmark, perform similarly in predicting the enantioselectivities of “unseen” ligands, when trained only on the “seen” historical data (Figure 6). One performance issue is the seen training set is highly biased toward ligands that give high enantioselectivities biasing the model’s predicted enantioselectivities. This may be the reason why the errors obtained in Figure 6 are slightly higher than those obtained in the “seen” set (Figures S4 and S5 distributions related to Figure 6). To test this idea, we separated the datapoints of the unseen set into higher selectivity and lower selectivity ligands, using a 90% *ee* threshold to categorize them. When doing this and calculating the metrics (see Figure S10A), we noticed that HCat-GNet improved to a MAE of 4.39 kJ mol^−1^ and RMSE of 5.49 kJ mol^−1^, while the Gradient Boosting experienced no change. Further training improves the fit (Figure S11).

**Figure 7:**
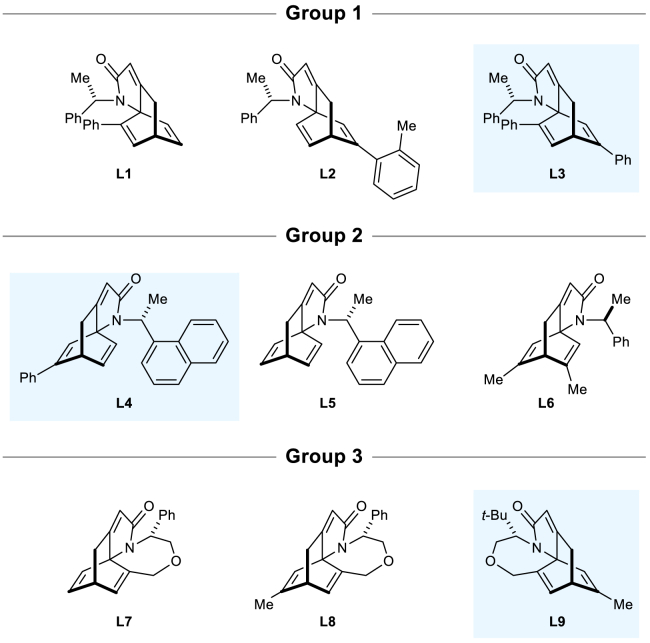
Groups of ligands analyzed for the “priority task” of selecting the most improved ligand (within the group) The real-world (experimental) best ligand (resulting in higher enantioselectivity) of each development ligand group is highlighted with a blue background. See main text for algorithm predicted order of selectivity.

The fact that the metrics of both models for this set are still higher than those obtained in the “seen” set even when only high selectivity reactions were considered made us investigate those datapoints where the error of the prediction was ≥5 kJ mol^−1^. Interestingly, we found that both methods failed to predict the selectivity of the same set of reactions, particularly substrates where the electrophilic carbon was not part of a ring, while reactions on cyclohex-2-en-1-one were always predicted with great accuracy. The reactions with higher errors were catalyzed by ligands that were used in other reactions with successful predictions. The same happened for the organoboron reagent, suggesting the presence of some (minor) mis-assignments in the experimental data may be present. Problematic substrates (Methods S3: analysis of problematic substrates, relates to Figures 6 and 7) were studied to understand this in more detail (Figure S15; Tables S1 and S2).

We focused on the performance of HCat-GNet (and its Gradient Boosting comparator) when predicting ligand driven RhCAA reaction enantioselectivities. As the training data is biased toward high selectivity, the results obtained in predicting the specific value of selectivity were poorer on low selective ligands (see Figure S10B). However, we found that even for ligands producing lower enantioselectivity, our method could successfully rank choices for future ligand improvement by defining a ligand “priority” task. Here we divided the ligands into three different groups, where differences in structure are given by small changes in functional groups (Figure 7).

For **Group 1** HCat-GNet ranks the three ligands in the correct order of engendered selectivity while Gradient Boosting only identifies the most enantioselective (**L3**). In **Group 2**, HCat-GNet identifies the most selective ligand **L4**, but fails to identify **L5** as the second most selective ligand. For this set, Gradient Boosting correctly predicted the order of enantioselectivity. In **Group 3**, HCat-GNet correctly identifies **L9** as the most selective catalyst, while Gradient Boosting could only identify **L7** as the least selective ligand. Thus, HCat-GNet was thus able to correctly identify the most enantioselective ligand in all cases. This suggests to us that HCat-GNet would have been an extremely useful tool in optimizing the structure of the Rit ligands^45^ and in minimizing the synthetic work required (Figure 1) (see Table S2 related to Figure 7).

**Figure 8:**
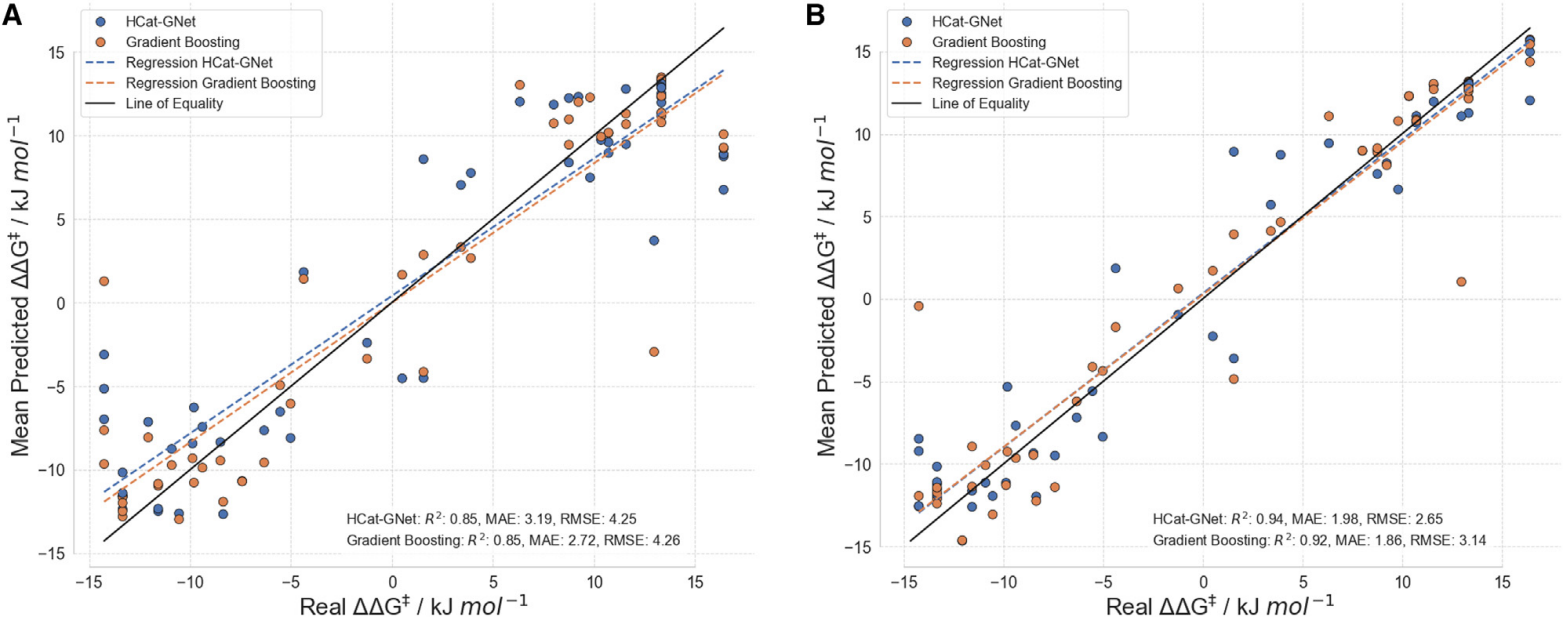
Testing HCat-GNet’s () ability to rank “unseen” ligand candidates (by ΔΔG^‡^ comparison) when hunting for new improved chiral ligands against Gradient Boosting () when using part of the “unseen” ligand data (A) Parity plot when keeping 50% of the “unseen” dataset^45^ outside of the nested cross-validation approach (A Wilcoxon test shows a *p*-value of <0.01 indicating the statistical difference between predictions of these methods, see Figure S11 for half included reactions in the nested cross-validation training and the half not-included), (B) Parity plot including all the “unseen” set^45^ in the nested cross-validation process (The Wilcoxon test *p*-value of <0.01 indicates a statistical difference between predictions of the two methods). Mean predicted values and standard deviations are available (Data S1).

As a final check, we asked both models to predict the enantioselectivity of the RhCAA reactions catalyzed by the Rit ligands when they had seen some of these ligands (a common position in chiral ligand development studies). To do so, first, we first added only half of the “unseen” reactions to the “seen” set and kept the other half as a final test. The nested cross-validation training approach was repeated and all the predictions of the original unseen set (contemplating both halves) were gathered to create the plot in Figure 8A. Lastly, we added all the 52 “unseen” reactions datasets to the original 668 “seen” reactions and applied the same procedure described before (Figure 8B). As shown in Figure 8, both the traditional and GNN approaches learn quickly, and that the performance of the predictions improves significantly. This suggests to us that HCat-GNet especially will learn at least as fast as the best Human experts in chiral ligand design, and its generality makes it a highly promising ligand development tool.

Additionally, we tried to predict the selectivity of these “unseen” ligands with CircuS to compare its performance against HCat-GNet (Figure S12B). Although CircuS representations are known to be very effective in interpolating chemical space and target variable, even when just a few datapoints are available,^22^ we found that its predictions on the “unseen” set are statistically worse than those of HCat-GNet, even though their performance was similar with the “seen” training set.

### Homogeneous catalyst graph neural network explainability

**Figure 9:**
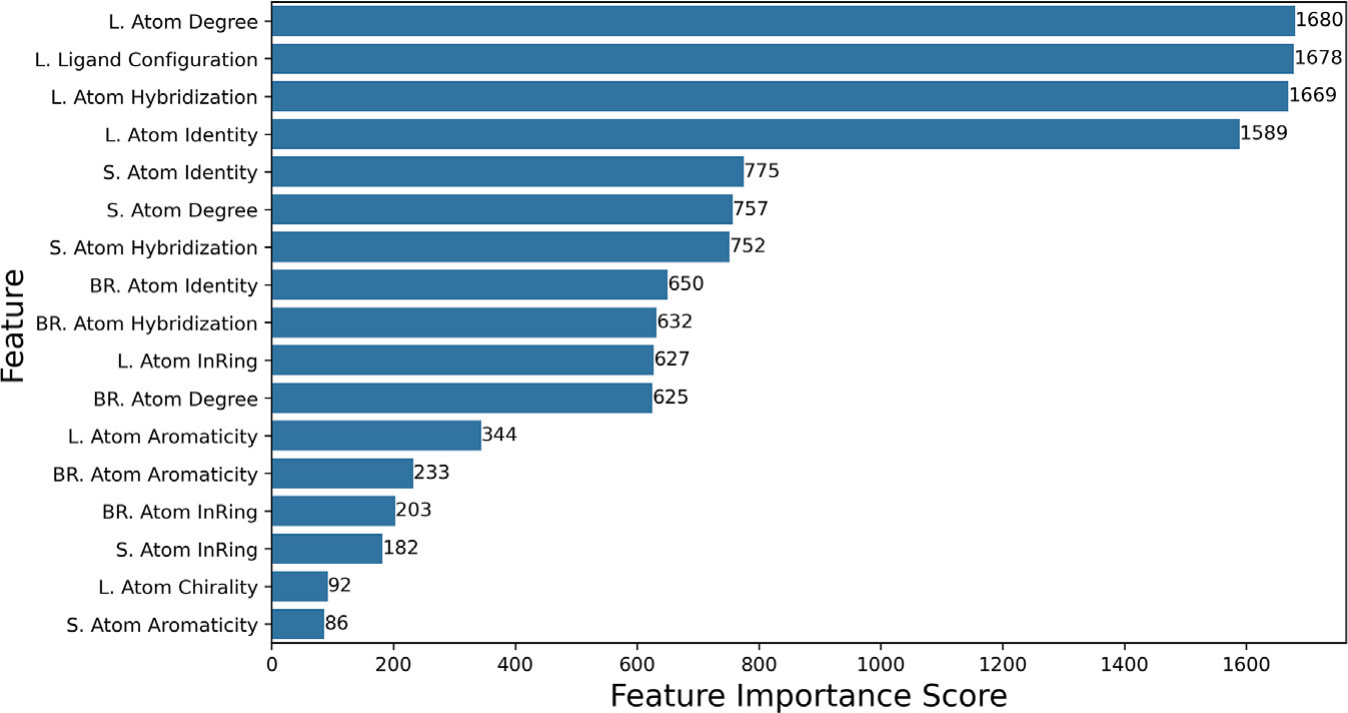
Attribution score of node features separated by molecule in enantioselective RhCAA reactions Here L symbolizes nodes (atoms) within the chiral diene ligand, S the substrate, and likewise BR the organoboron reagent (see also Method S4: graph denoiser tool and Figure S1 for node features).

The ability of Graph Neural Networks to analyze and correlate high dimensional-non tabular data to a target variable allows the use of explainable AI to understand explicit chiral ligand structure-enantioselectivity relationships. Thus, we applied the GNNExplainer to HCat-GNet to understand which node features in what molecules were most meaningful for the GNN to deliver accurate predictions. The results are shown in Figure 9.

Ligand atom degree (i.e., primary, secondary, tertiary substitution), followed by ligand configuration, atom hybridization, and atom identity are the highest rated attributes. We propose the atom degree feature is related to the steric factor of the node since atoms with a higher degree of substitution correspond to groups with higher volumes. Such steric analysis is also transferable to the hybridization of the ligand atoms, where atoms with sp^2^ and sp^3^ hybridization frequently correspond to high-volume groups, while sp hybridization indicates lower volume alkynes. In the case of ligand configuration, its high importance in combination with the classification metrics shown in Figure 4A demonstrates that the GNN is able to learn the correlation between the 3D descriptor and the facial selectivity of the process. The atom identity ranking indicates the GNN is able to understand the relation between the node identity and its effect on enantioselectivity. Because SMILES does not allow the representation of planar chiral nodes, the ligand atom chirality rating is artificially lowered. Fortunately, the HCat-GNet approach does allow planar chirality to be defined as a graph level feature (ligand configuration), which rates highly.

Atom identity, degree, and hybridization rank highly for the substrate but less so for the organoboron reagent. Other node features (e.g., aromaticity and atom in ring) across the reaction are found to be of lower relevance. This may be because such features can be derived from pre-existing node features as electronic, steric, and other effects are already learned within the training process.

**Figure 10:**
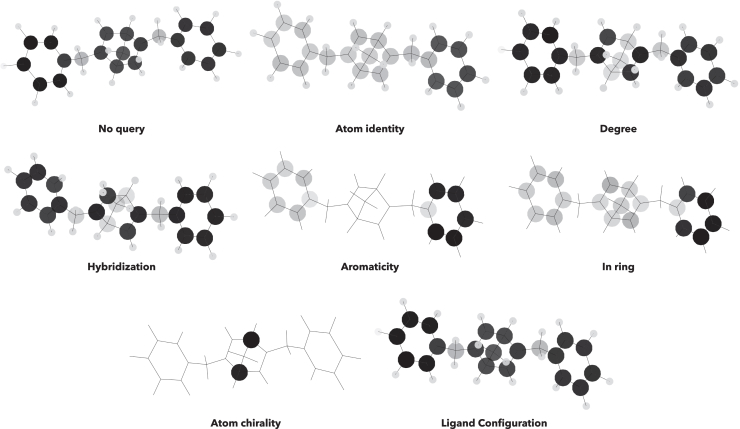
GNNExplainer ranking of seven types of ligand node feature to the atoms where this feature has the greatest effect on derived RhCAA reaction enantioselectivity Darker atoms (⋅) show locations where that node feature has a larger effect on ΔΔG^‡^ (reaction enantioselectivity).

GNNExplainer tools can be used to give information on regions within the ligand, substrate, or organoboron reagent that dominate reaction enantioselectivity. We created a software that converts the GNNExplainer node scores of selected node families (Figure S1) and maps them to a plot intensity for the molecule’s atoms (i.e., darker nodes are of high importance, transparent of little importance, to the ΔΔG^‡^ prediction). Figure 10 shows representative output from this approach.

From Figure 10 it is possible to make deductions of which node properties are more relevant at each position of a molecule. The tool exemplified in Figure 10 is a powerful synthetic chemistry aid as it highlights which regions of the ligand will be most profitable to make steric and/or electronic modifications. For example, in deciding between similarly sized *t*-Bu (vdW volume ∼74 Å^3^) and Ph (vdW volume ∼77 Å^3^) for potential new ligands are differentiated by the “aromaticity” and “in ring” features of Figure 10. It is also clear that at least one aryl unit is needed to maximize changes in reaction enantioselectivities (Figure 10 aromaticity plot). Potentially this may indicate a π-contact is present in the enantioselective step. Similarly, important analyses of the structures of any reaction component are possible with our tool, which is useful in overall reaction optimization. Further discussion of this approach is given (Methods S4: graph denoiser tool, relates to Figure 10, see also Figure S16).

**Figure 11:**
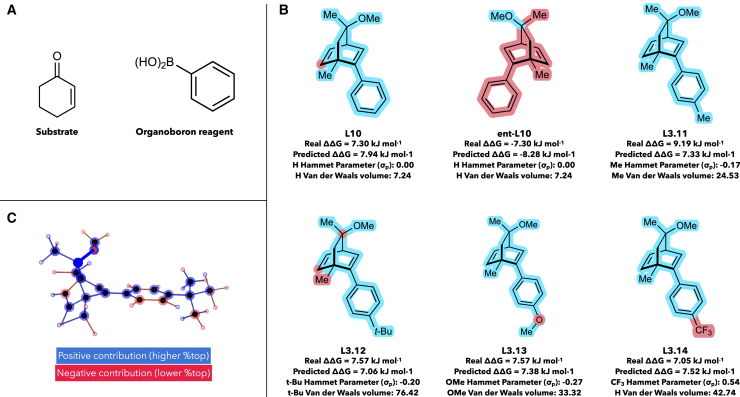
SHAP analysis of electronic effects in ligands with closely similar steric features (A) Reagents used (under identical reaction conditions) for each ligand. (B) Ligands analyzed for electronic effects. (C) Representative output of our software that visualizes the importance of each edge and node within a ligand to the final decision of the GNN Nodes and edges colored blue contribute to higher “%top” enantioselectivity and the converse for red features. The same for color coding is used in (B). van der Waals volumes were approximated using the Zhao method,^52^ and Hammett parameters were extracted from a review.^51^

To also understand substituent electronic effects within ligands, we used Shapley Value Sampling (SHAP) analyses. Although steric selection factors are well-understood in RhCAA reactions (e.g., Figure 2C),^21^ ligand electronic effects are not completely understood. HCat-GNet can provide understanding in this regard. Consider the identical RhCAA reactions of cyclohex-2-en-1-one and PhB(OH)_2_ (Figure 11A) catalyzed by six ligands (Figure 11B) differing in the identity of a substituent at the *para*-position of a phenyl ring. We created code that plots the ligand in 3D with colors depicting the contribution of each edge and node to the final decision of the GNN (Figure 11C).

Analysis of the enantiomers of **L10** confirms that the SHAP software developed does identify factors affecting enantiofacial selectivity. This agrees with the Hayashi stereochemical model,^21^ and shows the GNN is able to effectively model the effect of both relative and absolute ligand stereochemistry on reaction enantiofacial selectivity. While HCat-GNet does not exactly predict the RhCAA enantioselectivity for the ligands of Figure 11B, the order does follow that experimentally observed: Me, *t*-Bu, and OMe increase ΔΔG^‡^, and CF_3_ decreases it. SHAP analyses indicate that HCat-GNet can deduce electronic effects from its low-level node features. Electronically neutral **L10** (R_*para*_ = H) shows no electronic effects. Inductive electron-donating (+I) R_*para*_ groups (**L11**, Me and **L12** *t-*Bu) are identified as positive contributors to the ΔΔ G^‡^ while the CF_3_ (–I effect) group is a negative contributor. For **L13**, interestingly the GNN clearly indicates that inductive effects are potentially more important in the RhCAA reaction as it classifies the oxygen as a negative (-I) contributor toward enantioselectivity, rather than as a (+M) OMe unit.

Clearly the Shapley Value Sampling (SHAP) methods exemplified in Figure 11 are much more information-rich than the simple steric stereochemical model of Hayashi (Figure 2C).^25^ By combining this with the GNNExplainer software (exemplified in Figures 9 and 10), extraction of all reaction component features needed for high enantioselectivity in the RhCAA reaction is possible. Although exemplified here for RhCAA reactions, the HCat-GNet can, without the modification of its code, be used for any ML∗-catalyzed asymmetric process. Thus, it can be used in the explainable AI co-pilot role imagined for the ligand optimization process of Figure 1.

### Homogeneous catalyst graph neural network transferability

**Figure 12:**
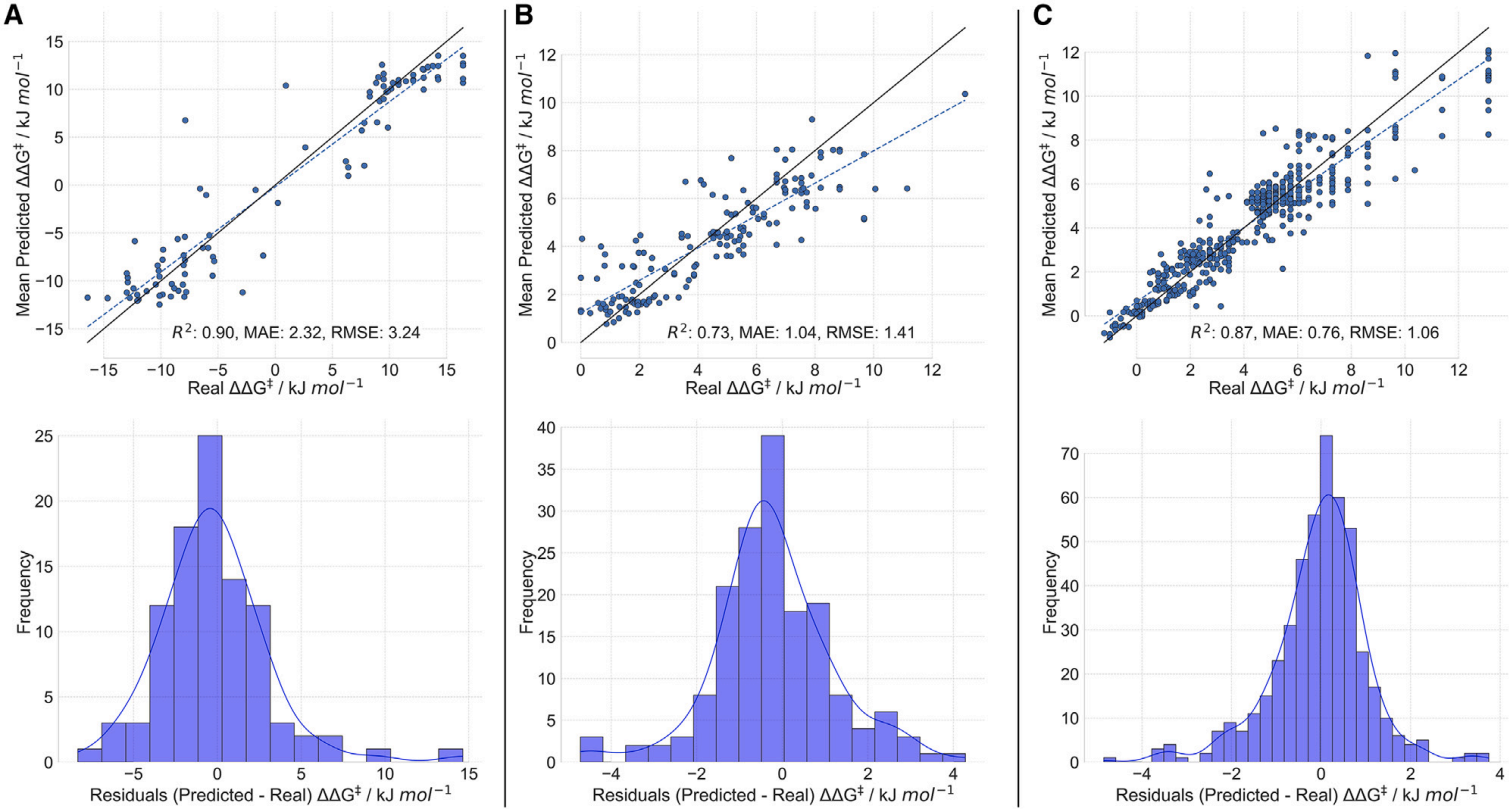
Evaluation of performance of HCat-GNet on other asymmetric reactions Parity plot and residuals distribution of HCat-GNet predictions of the “unseen” dataset for: (A) the BiAryl ligand RhCASA dataset (split in an 8:2 train/test ratio), (B) the CADA reaction using hypervalent iodine(III) ligands dataset (split in an 8:2 train/test ratio), and (C) for asymmetric *N*,*S*-acetal formation using chiral phosphoric acids (split into 600 points for training and 475 for the test).

We confirmed HCat-GNet as a straight forward general approach by modeling three additional chiral ligand controlled asymmetric catalytic reactions. First, we applied HCat-GNet to new analyses of RhCASA reactions catalyzed by chiral diphosphine ligands (Methods S5: generalization, relates to Figure 12) to show our method can model axial ligand chirality from SMILES inputs (Figure 12A). Secondly, we undertook HCat-GNet analysis of published Catalytic Asymmetric DeAromatisation (CADA) reactions,^16^ showing effective modeling (Figure 12B) of chiral spirocentre iodine(III) ligands. HCat-GNet’s effectiveness is competitive with the published DFT-based feature approach.^16^ Finally, we applied HCat-GNet to Denmark’s asymmetric *N*,*S*-acetal forming reaction/dataset (Figure 12C).^26^ The graph representations of all three reactions are rapidly accessible by simple workflows analogous to those used in the diene ligand RhCASA datasets. For the BiAryl dataset only change of the ligand configuration feature from planar to axial chirality. For the CADA dataset, only features representing the reagent concentrations were added to the molecular graphs. For the thiol additions, essentially identical approaches to those of the RhCASA study were used. Only very minor feature modifications were needed in each case to account for these three diverse reactions. We split each of these datasets into a “seen” and “unseen” sets randomly, applied a nested cross-validation approach to the seen set, and predicted the values of the unseen set the same way we did with the diene database. We calculated the mean of each “unseen” point and gathered the results in a single plot, shown in Figure 12.

As the BiAryl dataset is previously unanalyzed we also subjected it to CircuS^22^ descriptors (see Figure S13). HCat-GNet performs better than CircuS by RMSE for both seen and unseen sets, but only for the latter is a statistical difference found. For the literature CADA dataset, the HCat-GNet RMSE is within 0.32 kJ mol^−1^ of that of Gao.^16^ Although the different train/split strategies of the two approaches make direct comparison challenging, it is possible to say that HCat-GNet’s predictions are of high accuracy for this challenging set without recourse to any DFT calculations. Lastly, for the Denmark thiol addition dataset, HCat-GNet attained an RMSE of 1.06 kJ mol^−1^ and an MAE of 0.76 kJ mol^−1^. Other articles report models for this dataset, including Gong et al.^15^ attaining a mean MAE of 0.40 kJ mol^−1^ in the cross-validation process (5-folds), Sandfort et al.^17^ with a MAE of 0.60 kJ mol^−1^ using random train/test split (600/475), Denmark and coworkers^26^ with a MAE of 0.64 kJ mol^−1^ (random 600/475 train/test split), and Li et al.^40^ (method that relies in DFT in combination with GNNs) with a MAE of 0.82 kJ mol^−1^ (random 600/475 train/test split). Again, the different splitting strategies make direct performance comparisons challenging. However, it is clear that HCat-GNet is still a very useful initial tool for chiral ligand development, especially as its interpretability tools are readily usable by synthetic chemists without specialist informatics knowledge (HCat-GNet has also been successfully applied to data curation and ligand study in catalytic asymmetric decarboxylative allylation. Additionally, a help interface [usable by non-informatics specialists] is in development. All these aspects will be reported on in the near future).

## Discussion

HCat-GNet consists of a graph neural network model predicting the stereoselectivity and absolute stereo configuration of the major product of any asymmetric reaction accelerated by any metal-ligand complex ML∗ (L∗ = any chiral ligand). We demonstrate its applicability by predicting the enantioselectivity of >500 experimental RhCAA reactions using only the SMILES representation of the reacting molecules as inputs. HCat-GNet is competitive with state-of-the-art current machine learning approaches^26^ without having to curate/DFT calculate bespoke descriptors. HCat-GNet also produces human-understandable outputs useful for improvement in new ligand design. When challenged with a test set of “unseen” ligands resulting from a genuine ligand development project,^38^ HCat-GNet is able to identify which features of the chiral ligand would have the strongest effects on improved enantioselectivity at least as well as human experts in ligand design. Finally, unlike DFT-based mechanistic approaches, HCat-GNet can be used to optimize a broader range of ML∗-catalyzed processes even when the mechanism is not known. Finally, the fact that HCat-GNet is agnostic to the reaction type means it has widespread potential application in asymmetric catalysis.

### Limitations of the study

In its present form, HCat-GNet interrogates selectivity effects caused by the complex’s chiral ligand (L∗) alone, rather than the metal catalyst (ML∗) as a whole. This has not raised any issues herein, but the potential that a more detailed graph representation of the whole catalyst might be needed in some cases should be noted.

## Resource availability

### Lead contact

Requests for further information and resources should be directed to and will be fulfilled by the lead contact, Grazziela Figueredo (g.figueredo@nottingham.ac.uk).

### Materials availability

This study did not generate new unique reagents.

### Data and code availability

• The datasets generated during this study are available at Zenodo and GitHub and are publicly available at the date of publication. The URL is listed in the key resources table.
• All original code has been deposited at Zenodo and GitHub and is publicly available at of the date of publication. The URL is listed in the key resources table.
• Any additional information required to re-analyze the data reported in this article is available from the lead contact upon request.

## Acknowledgments

We acknowledge funding from the Doctoral Training Centre in Artificial Intelligence of the School of Computer Science of the 10.13039/501100000837University of Nottingham. The Engineering and Physical Sciences Research Council (EPSRC) for grant EP/S035990/1 (Accelerated Discovery and Development of New Medicines: Prosperity Partnership for a Healthier Nation).

## Author contributions

Conceptualization, E.A.B., E.Ö., S.W., and G.F.; methodology, E.A.B., E.Ö., S.W., and G.F.; software, E.A.B.; investigation, E.A.B., E.Ö., S.W., and G.F.; resources, R.K.R., H.L., and J.C.M.; data curation. E.A.B.; writing – original draft, E.A.B. and S.W.; writing – review and editing, E.Ö., H.W.L., and G.F.; supervision, E.Ö., H.W.L., S.W., and G.F.

## Declaration of interests

A patent (WO2024084227A1) associated with the synthetic aspects of this work is held by the University of Nottingham naming S. Woodward, H. Y. Lam and R. K. Rit as inventors. There is no other interest to declare.

## STAR★Methods

### Key resources table

**Table:** 

REAGENT or RESOURCE	SOURCE	IDENTIFIER
**Deposited data**		

RhCASA ‘seen’ curated dataset	Open Source	GitHub: https://github.com/EdAguilarB/hcatgnet/tree/core/data/datasets/rhcaa/known_unknown/learning/raw/rhcaa.csv
RhCASA ‘unseen’ curated dataset	This paper	GitHub: https://github.com/EdAguilarB/hcatgnet/tree/core/data/datasets/rhcaa/known_unknown/test/raw/rhcaa.csv
BiAryl curated dataset	This paper	GitHub: https://github.com/EdAguilarB/hcatgnet/blob/core/data/datasets/biAryl/biaryl.csv
CADA dataset	Open Source	GitHub: https://github.com/FlybenChemEngineer/ML_ICat/blob/main/Dataset/ICatDataset1128.xlsx
Asymmetric *N*,*S*-acetal forming reaction dataset	Open Source	https://github.com/Shuwen-Li/SEMG-MIGNN/blob/main/Data/data2/data2.csv
Metrics, statistical analyses, all data for all runs	Open Source	Data S1: Wilcoxon statistical data, related to all data containing Figures (.zip)

**Software and algorithms**		

HCat-GNet (all, self-contained, software code needed to run analyses)	This paper	Zenodo: https://zenodo.org/records/13954130

### Method details

#### Molecular representations

##### Bespoke descriptors

The bespoke descriptor calculation procedure was directly taken from ref. 20. To do this, we identified all the substituents within the ligand structure and calculated their van der Waals volume using Zhao’s method,^52^ while electronics were captured by their Hammet parameter in a review.^51^

##### CircuS

The CircuS descriptors were calculated as they were in their original implementation.^22^ For that, we used the DOPtools library, a lower limit of 0 and an upper limit of 2 in the ChytonCircus function. All the generated descriptors from the learning set were the ones used for modeling. Descriptors found in the ‘unseen’ set that were not found in the ‘seen’ set were not included, while descriptors not found in the ‘unseen’ set that were found in the ‘seen’ set were given a value of 0 for all reactions within the unseen set.

##### Graph representation

The graph representation was created using RDKit. To do this, the algorithm iterates through all the participant molecules. For each molecule, the adjacency matrix is obtained and transcribed into an adjacency list. The node feature matrix is created by using RDKit’s built-in functions. The configuration feature and temperature of reaction were hardcoded in the.csv file, and the algorithm was design to take such values and incorporate them into the graph representation.

###### Ligand configuration feature

We decided to add a reaction-level feature to the graph representation. This feature identifies the planar chirality of the ligand, as the SMILES representation of the ligand itself does not carry such information (see Figure S3). Even though the atom chirality feature implicitly encodes the configuration of the ligand, we found that adding this feature as a system-level feature increased the performance of the model as it implicitly carries the same information that would result from assigning the planar chirality to the coordinated C=C atoms (nodes) in the active ligand.

#### Machine learning algorithms training

##### Traditional machine learning approaches

Gradient boosting, random forest, and linear regression models were trained using Scikit-learn. Hyperparameter were tuned using the whole ‘seen’ set from a dictionary of possible values with built-in functions from Scikit-learn.

##### Graph Neural Network

All GNN models were created and trained using the pytorch geometric. Raytune was used for hyperparameter optimisation. Adam was used as optimiser with a learning rate of 0.01 was used. ReduceLROnPlateau was used as scheduler, with a patience of 7 epochs. The validation set was used to save the best model based on the loss for this set, and also for early stopping purposes.

###### Model architecture

Each graph consists of nodes that represents the atoms, and edges that represent covalent bonds. The components of the RhCAA catalytic reaction (ligand, substrate, organoboron reagent) were described as separate graphs, which were further concatenated to represent the whole reaction system (Figure S2). Each node contains the information on atom identity, atom degree (number of connected bonds to adjacent non-hydrogen atoms), atom hybridisation, if the atom is part of an aromatic system or not, if the atom is part of a ring or not, atom center of chirality (*R, S,* or none), and the graph-level variable ligand configuration (to represent the ligand’s planar chirality). These features were used to generate a one-hot-encoded atom representation vector of final length 24 (see Figure S1). The following workflow transforms a given graph input into an internal energy value.

(1) The node features are taken (length of 24) and are expanded to a final length of 64 by a graph convolutional operator with leaky ReLU activation function, R^nodes×24^ → R^nodes×64^.
(2) The graph convolution operator updates all the node states in parallel to update the nodes features to graph-aware features once with leaky ReLU activation function, R^nodes×64^ → R^nodes×64^.
(3) Mean and max pooling is applied elementwise to all the node feature vectors to get a graph-level feature vector, R^nodes×64^ → R^1×128^.
(4) A fully connected layer with leaky ReLU activation function takes the graph-level vector and maps it to half of its length, R^1×128^ → R^1×64^.
(5) A last fully connected layer transforms the feature vector into a scalar number, this being the prediction of the model, R^1×64^ → R^1^.

###### Model training

For the training, an Adam optimizer was used, using the root mean squared error as the loss function. In each epoch, the loss of the validation set was supervised for two reasons: learning rate adjustment and early stopping. In cases where there was no improvement in the validation loss for 5 epochs, the learning rate was multiplied by a factor of ×0.7. At the beginning of the training, the learning rate was set to 0.01, and the minimum learning rate allowed was set to 10^−8^. In case that there was not improvement in the validation set for 30 epochs, then the training was stopped. For the learning process, the training set was fed to the model in batches of 40 graphs, the loss and gradients were calculated for each batch, and weights and biases updated after each batch. In each epoch, all the structures of the training set were fed into batches, and an epoch finalised once all batches had gone through the backpropagation process. To assess robustness and generalisation of the models, we have applied an inner cross validation strategy. This splitting strategy consists in dividing the dataset into *k* folds and utilise each fold as test set, but also in each test set, each of the remaining *k-1* folds will be used as validation set once, while the remaining *k-2* folds will be used as training set. This way, for each test fold, a total of *k-1* training different processes will be performed, each with different training and validation sets. By doing this, it is possible to analyze the model generalisability when training on different datapoints. For our case, since we have created 10-folds, there would be a total of 10 test sets, while each test seat will have 9 different training processes using different validation and training sets, leading to a total of 90 different training processes.

###### Model explainability

We used GNNExplainer implemented in Torch Geometric 2.4.0 to explain node features. This algorithm assigned a score to each node feature of each node. We have summed the scores of those related features (i.e., features with the same color code, see Figure S1) for each molecule in the reaction separately. This allowed us to analyze the importance of overall features in each molecule instead of individual one-hot-encoded specific features for the reaction-graph representation.

For Shapley Sampling Value, implemented in Torch Geometric 2.4.0, we explained the impact of specific nodes in the outcome of the asymmetric reaction. The scores of each node were normalised from −1 to +1, and then we colored the atoms from red (−1) to blue (+1).

###### **ΔΔ**G^‡^ target variable calculation

The measured values of selectivity of the different reactions within our two databases were reported in terms of the enantiomeric ratio and enantiomeric excess. To transform this variable into a ΔΔG^‡^ value, we first converted the enantiomeric excess values to enantiomeric ratio using the Equation 3.

(Equation 3) $$\text{er}=\frac{\left(\text{ee}+100\right)}{2}\cdot 100$$

The *er* of the ‘bottom’ isomer was obtained. Then, we assumed chemical equilibrium of the transition states leading to the two possible enantiomeric products of the reaction, defined by Equation 4.

(Equation 4) $$K=\frac{er_{\text{bottom}}}{100-er_{\text{bottom}}}$$

Lastly, to attain the Gibbs free energy, we took the K values and used Equation 5.

(Equation 5) $$\Delta \Delta G^{‡}=-\text{RTln}\left(K\right)$$

### Quantification and statistical analysis

For the calculation of statistical differences, we made use of the Wilcoxon test as available in the stats module of the package SciPy of Python. This function takes two inputs ‘x’ and ‘y’, and tests the null hypothesis that these two paired samples come from the same distribution. It determines if the distribution of the differences ‘x-y’ is symmetric about zero. This is a non-parametric version of the paired *t-test*.^54^ Wilcoxon statistical tests were conducted when comparing the effectiveness of HCat-GNet against other informatics approaches. Data for all tests are available (Data S1: Wilcoxon statistical data).

## Supplemental information

Document S1. Figures S1–S16, Tables S1–S4, and Methods S1–S5

Data S1. Raw results of HCat-GNet performance against traditional machine learning approaches for all the reactions analyzed in this study.
