# Supplementary material for: Homogeneous catalyst graph neural network: A human-interpretable graph neural network tool for ligand optimization in asymmetric catalysis
Source: iScience. 2025 Jan 23;28(3):111881. doi: 10.1016/j.isci.2025.111881 (PMC11889620; doi:10.1016/j.isci.2025.111881)
Supplement: Document S1. Figures S1–S16, Tables S1–S4, and Methods S1–S5 [file mmc1.pdf]

iScience, Volume *28*

Supplemental information

Homogeneous catalyst graph neural network: A human-interpretable graph neural network tool for ligand optimization in asymmetric catalysis

Eduardo Aguilar-Bejarano, Ender Özcan, Raja K. Rit, Hongyi Li, Hon Wai Lam, Jonathan C. Moore, Simon Woodward, and Grazziela Figueredo

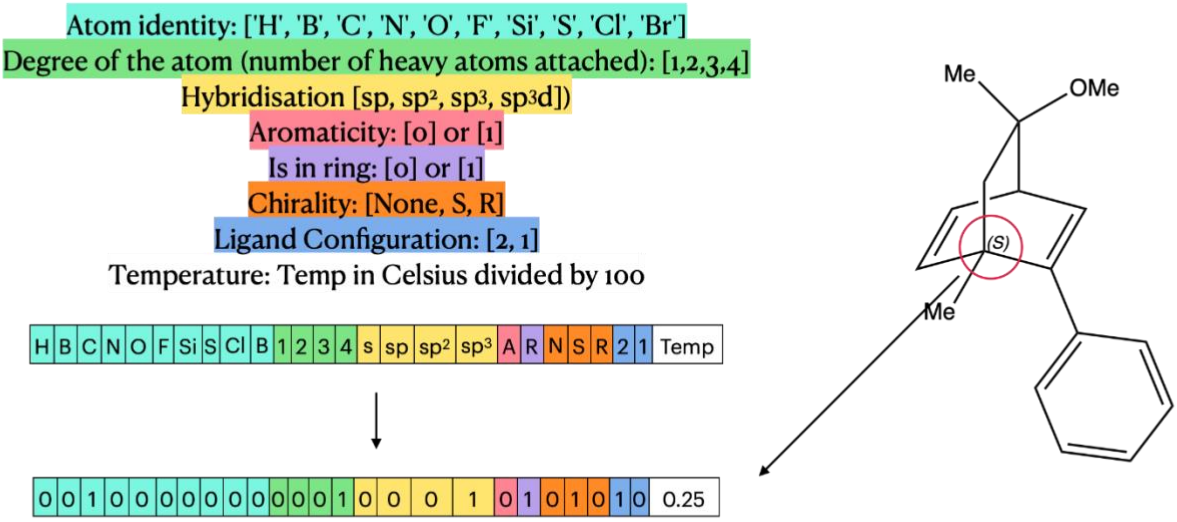

Figure S1. Illustrative exemplar of node features applied to one atom within a representative ligand **L10** (in our main paper), structure **L3.40** in primary literature (see Reference S1 and **Table S4**). The resultant one-hot-encoded vector of **L10** is shown.

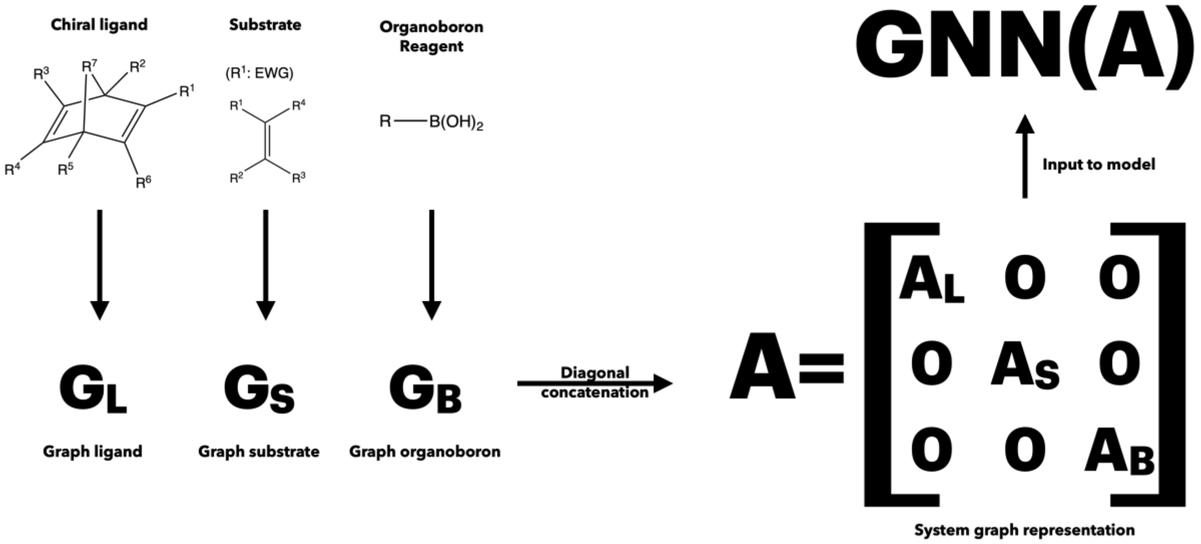

Figure S2. Scheme of the reaction system representation used as the input for the GNN models.

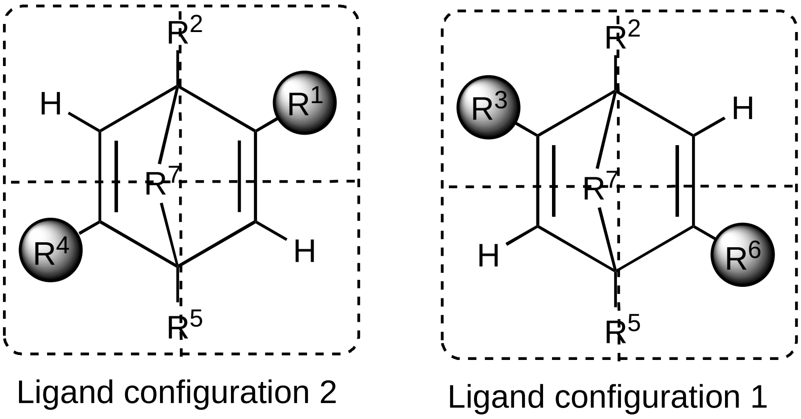

Figure S3. Examples of the criteria followed to add an overall chiral ligand configuration feature to the reaction graph representation using a quadrant model to encode the planar chirality of the ligand.

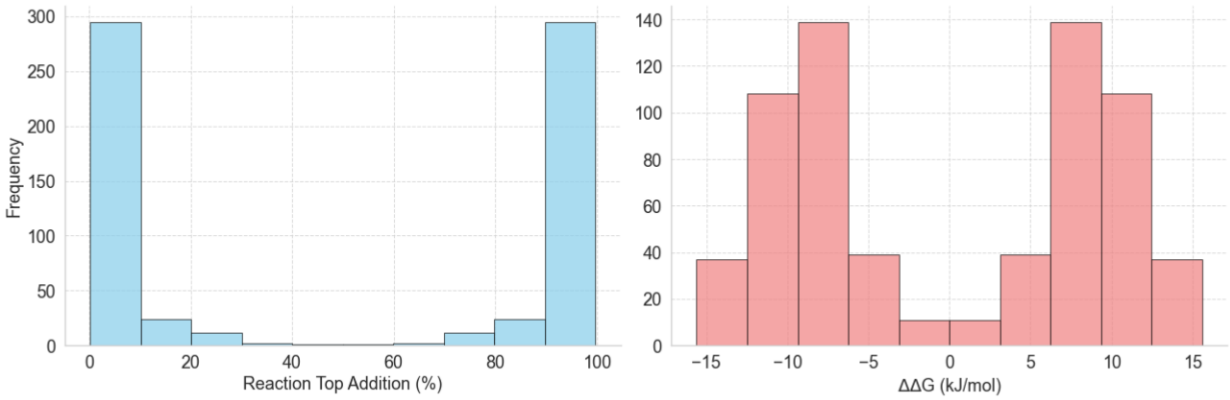

Figure S4. Distribution of values of %top and ΔΔG‡ target variable in the 'seen'[S1-S2] data set.

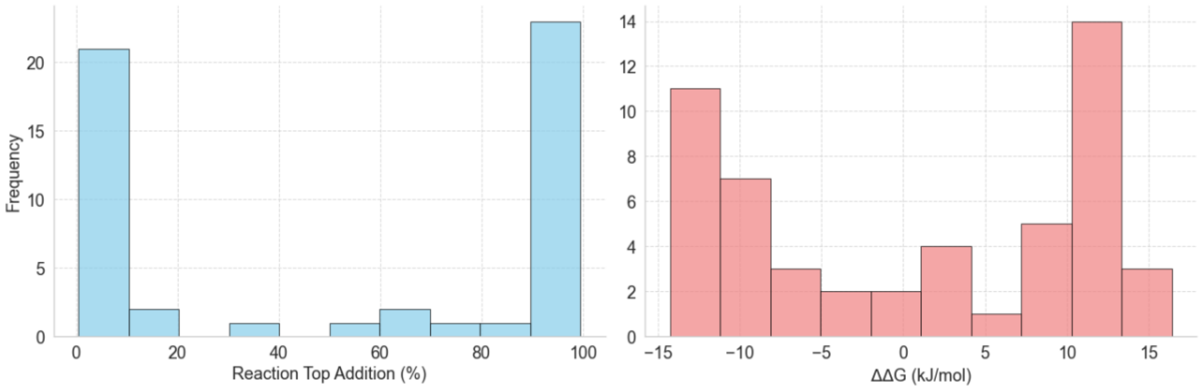

Figure S5. Distribution of values of %top and ΔΔG‡ target variable in the 'unseen' set (see References S1-S4 and Tables S3-4).

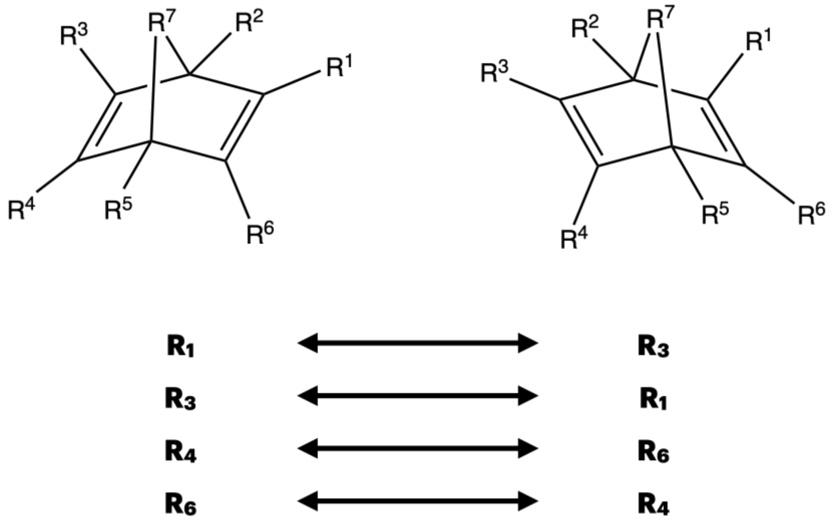

Figure S6. Change or ordering of carbons in the 1,3-cyclohexadiene depending on the configuration of the ligand and equivalent positions of R^n (n = 1-6) changes between a "real" and a "virtual" (enantiomeric) ligands (Methods: S1).

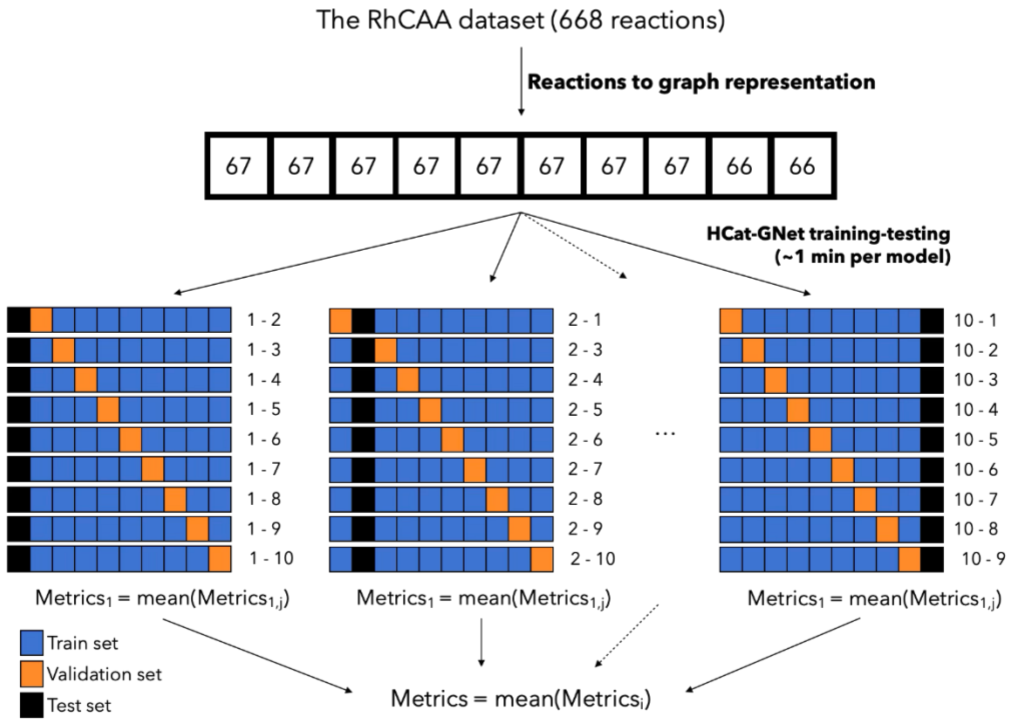

Figure S7. Scheme of the inner cross validation approach used for data splitting applied in this study.

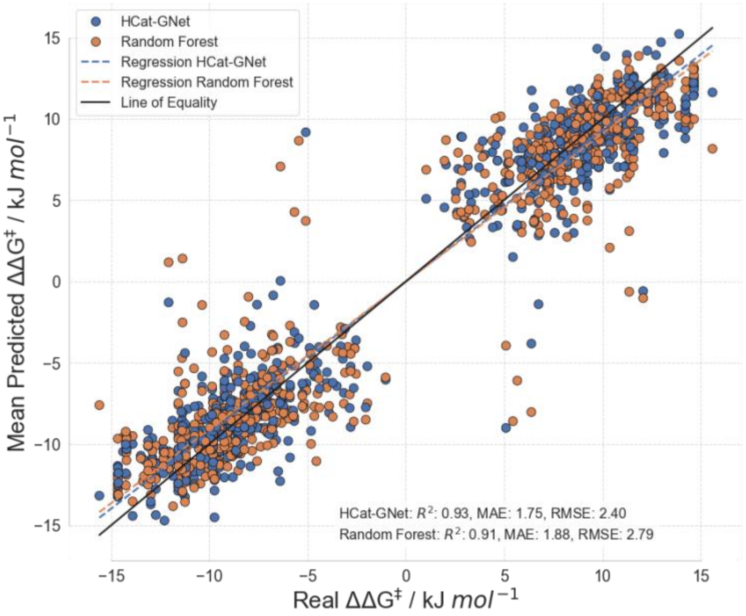

Figure S8. Parity plot of the mean predicted ΔΔG‡ by the 9 different training processes for each test point and the experimental ΔΔG‡ using the Random Forest algorithm and the bespoke descriptors.

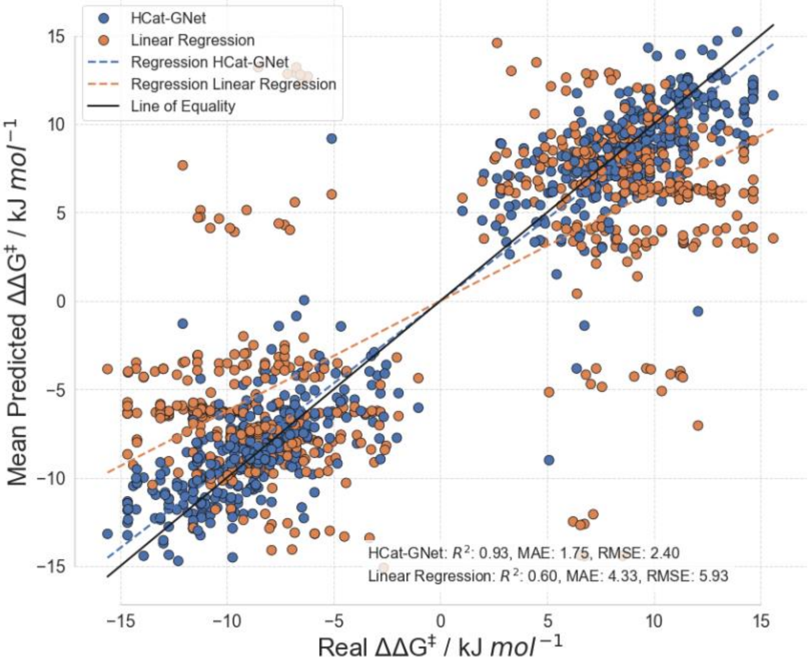

Figure S9. Parity plot of the mean predicted ΔΔG‡ by the 9 different training processes for each test point and the experimental ΔΔG‡ using the Linear Regression algorithm and the bespoke descriptors.

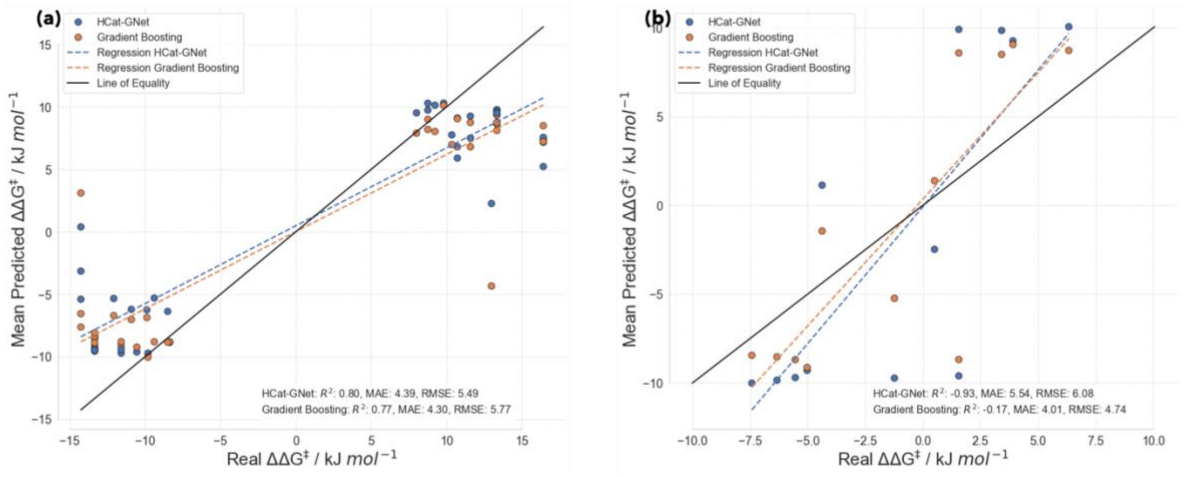

Figure S10. Parity plot of mean predicted ΔΔG‡ and the experimental ΔΔG‡ for (a) high selective development ligands (**L1a**, **L3a**, **L3b**, **L15-17**, **L19**, **L20**, **L21**) and (b) low selective development ligands (**L1b**, **L2a**, **L2b**, **L4a**, **L4b**, **L5-9**, **L18**). See also Tables S3-4. To separate the plots and statistics into two groups, we chose a 7.5 kJ mol^{-1} threshold (equivalent to 90% *ee* for these reactions). Reactions that exhibit higher selectivity than the 90% *ee* threshold, **(a)**, were gathered and subjected to separate statistics in this plot. The same procedures were applied to the low selectivity group ((<90% *ee* **(b)**).

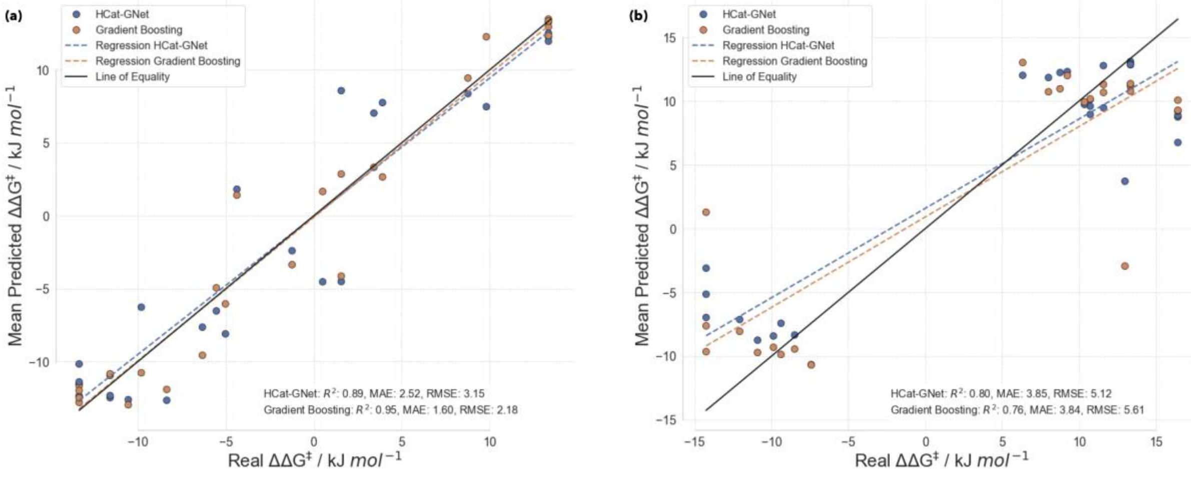

Figure S11. Parity plot of mean predicted ΔΔG‡ and the experimental ΔΔG‡ for the 'unseen' dataset when half of the points are introduced to the nested cross validation approach and half are kept completely unseen comparing HCat-GNet (●) and Gradient Boosting (●) using Owen's[S1] approach. **(a)** Shows all the 'unseen' data points that were introduced into the nested cross validation training approach, **(b)** shows the 'unseen' datapoints that were kept outside the nested cross validation.

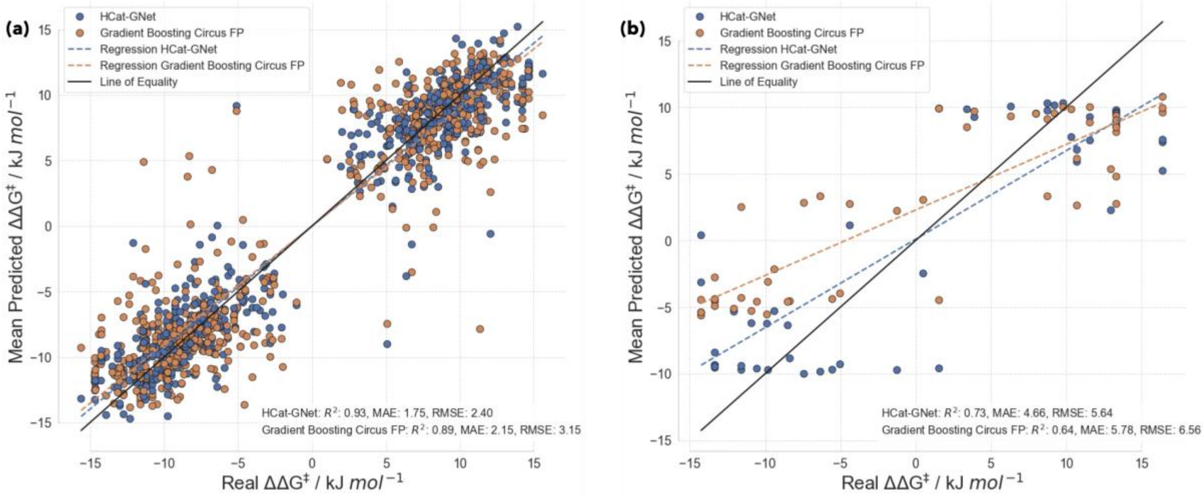

Figure S12. Parity plot of the mean predicted ΔΔG‡ by the 9 different training processes for each test point and the experimental ΔΔG‡ comparing HCat-GNet (●) predictions against Gradient Boosting using CircuS Fingerprint descriptors (●). **(a)** Parity plot for the 'seen' set (no significant difference by Wilcoxon test, p-value 0.338) and **(b)** Parity plot for the 'unseen' set (significant difference by Wilcoxon test, p-value <0.001), worse performance with CircuS features.

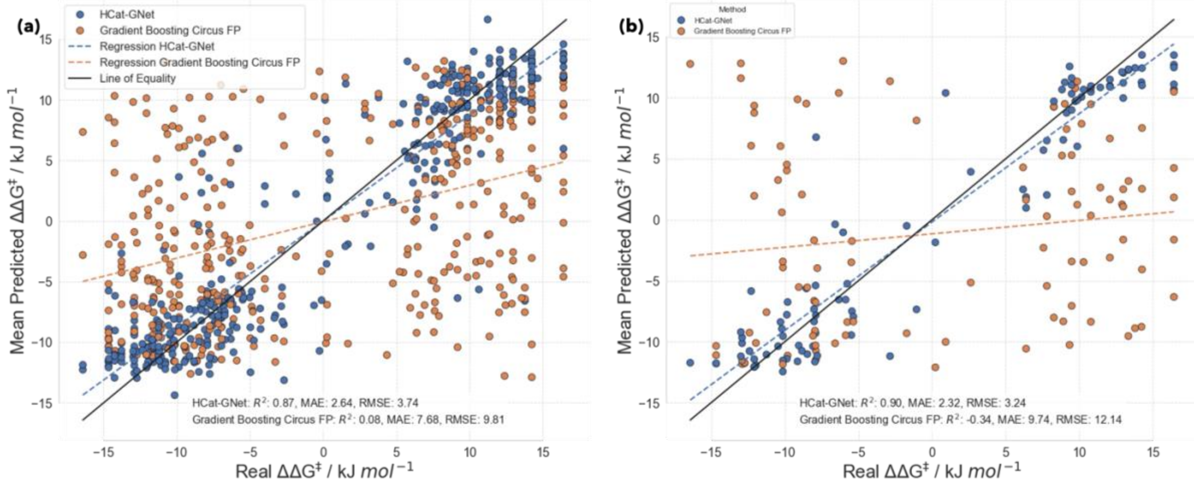

Figure S13. Parity plot of the HCat-GNet (●) and the Gradient Boosting with CircuS Fingerprints (●) of the mean predicted ΔΔG‡ from the nine different training processes for each test point and the experimental ΔΔG‡ for the additional BiAryl dataset (see Methods: S5). **(a)** Shows the results for the 'seen' set (A Wilcoxon test shows a p-value of 0.838, showing no statistical difference, but with different RMSE values) and **(b)** shows the results for the 'unseen' set (A Wilcoxon test shows a p-value < 0.001, indicating statistical difference between predictions of the methods).

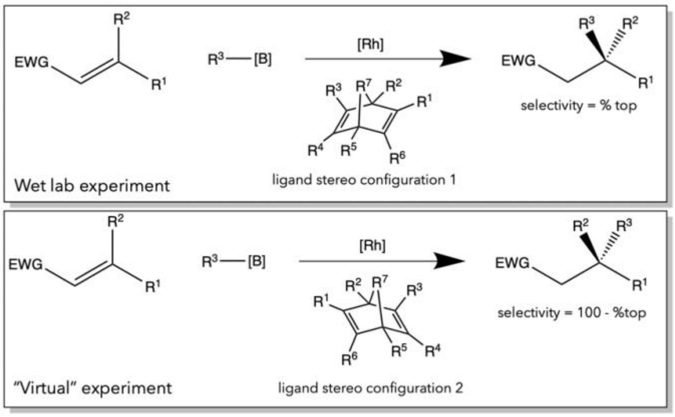

Figure S14. Virtual conversions to augment and balance the training dataset (References S1-S2) used.

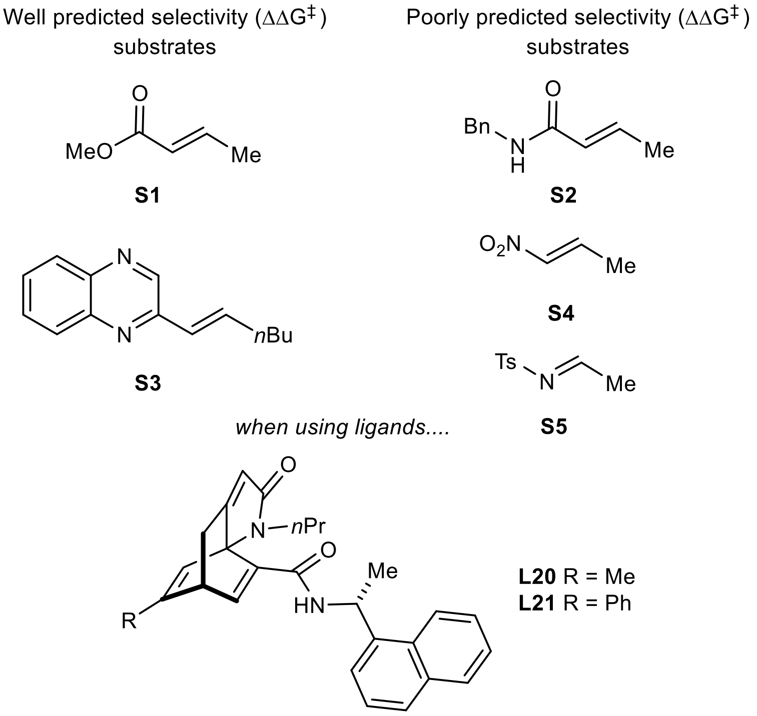

Figure S15. Related substrates structures where both HCat-GNet and Gradient Boosting perform poorly vs. well behaved substrates.

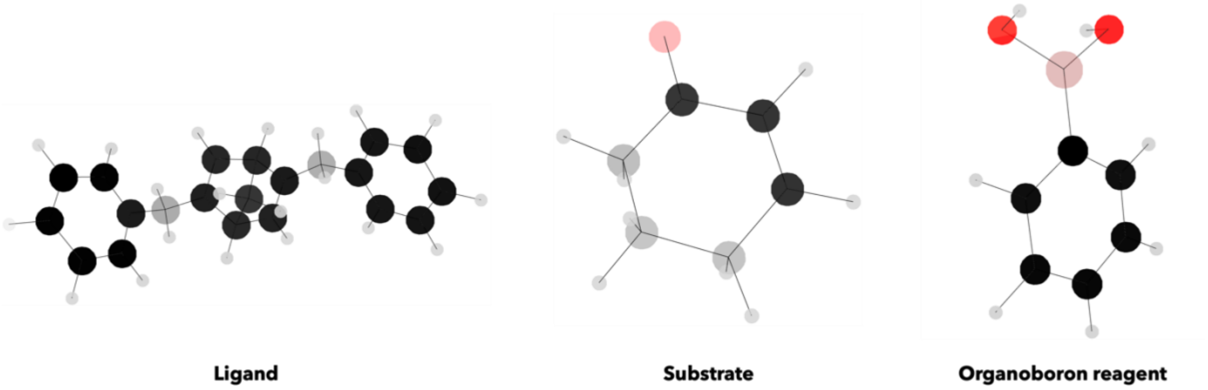

Figure S16. Denoised reaction graph after using the GNNExplainer algorithm and use the scores obtained per node as transparency values (higher importance = higher score = darker atoms).

Table S1. Tabulated data of experimental and predicted ΔΔG‡ for reactions where both methods had an error ≥5 kJ mol^{-1} in the 'unseen' set.

Reaction Index	Substrate[a]	Ligand	Experimental ΔΔG‡/ kJ mol^{-1}	HCat-GNet ΔΔG‡/ kJ mol^{-1}	Gradient Boosting ΔΔG‡/ kJ mol^{-1}
22	**S2**	**L20**	-14.26	0.39	3.11
23	**S2**	***ent*-L20**	12.98	2.27	-4.34
24	**S4**	**L20**	-14.26	-3.14	-7.62
25	**S4**	***ent*-L20**	16.42	5.22	8.50
30	**S5**	**L20**	-14.26	-5.40	-6.55
31	**S5**	***ent*-L20**	16.42	7.37	7.16
32	**S5**	**L21**	-12.07	-5.34	-6.70
33	**S5**	***ent-L21***	16.42	7.57	7.23

[a] Structures given in Figure S15.

Table S2. Tabulated data of experimental *ee*, real ranking of selectivity, and the machine learning given ranking to different families of ligands depicted in Figure 7.

**Group**	Ligand	Observed *ee*	Real selectivity ranking	HCat-GNet selectivity ranking	Gradient Boosting selectivity ranking
1	**L1**	94	**2**	**2**	3
	**L2**	65	**3**	**3**	2
	**L3**	96	**1**	**1**	**1**
2	**L4**	80	**1**	**1**	**1**
	**L5**	70	**2**	3	**2**
	**L6**	10	**3**	2	**3**
3	**L7**	24	**3**	2	**3**
	**L8**	30	**2**	3	1
	**L9**	59	**1**	**1**	2

Table S3. Enantiomeric excesses of reactions reported herein used in the new 'unseen' ligand set (see also Methods S2: preparation and use of 'unseen' ligands).

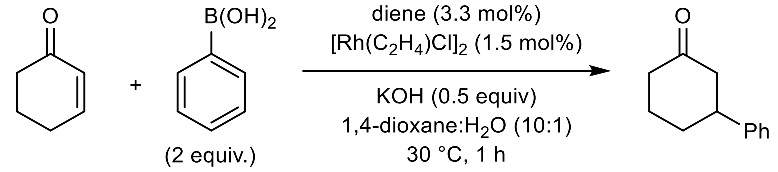

Ligand	Enantiomeric Excess (*ee*, %)	Product absolute stereochemistry (Label in Reference S1)
**L1a**	94	*S* ('top'[S1])
**L1b**	85	*R* ('bottom'[S1])
**L2a**	65	*S* ('top'[S1])
**L2b**	76	*R* ('bottom'[S1])
**L3a**	96	*S* ('top'[S1])
**L3b**	96	*R* ('bottom'[S1])
**L4a**	30	*S* ('top'[S1])
**L4b**	80	*R* ('bottom'[S1])
**L5**	70	*R* ('bottom'[S1])
**L6**	10	*R* ('bottom'[S1])
**L7**	24	*S* ('top'[S1])
**L8**	30	*S* ('top'[S1])
**L9**	59	*S* ('top'[S1])

**L15**	95	*S* (‘top’[S1])
**L16**	92	*S* (‘top’[S1])
**L17**	94	*S* (‘top’[S1])
**L18**	85	*S* (‘top’[S1])
**L19**	90	*R* (‘bottom’[S1])
**L20**	97	*R* (‘bottom’[S1])
**L21**	>99	*R* (‘bottom’[S1])

Table S4. Ligand primary literature sources (see also Methods S2: preparation and use of ‘unseen’ ligands).

Ligand identifier (this paper)	Literature Source (ref)	Ligand identifier in literature source quoted
**L1a,b**	Hongyi Li Thesis[S4]	**9**
**L2a,b**	Hongyi Li Thesis[S4]	**10**
**L3a,b**	Hongyi Li Thesis[S4]	**11**
**L4a,b**	Hongyi Li Thesis[S4]	**13**
**L5**	Hongyi Li Thesis[S4]	**12**
**L6**	This paper	**L6**
**L7**	Hongyi Li Thesis[S4]	**20a**
**L8**	Hongyi Li Thesis[S4]	**20b**
**L9**	This paper	**L9**
**L10**	Burns *et al.* review[S2]	**L3.40**
**L11**	Burns *et al.* review[S2]	**L3.46**
**L12**	Burns *et al.* review[S2]	**L3.47**
**L13**	Burns *et al.* review[S2]	**L3.50**
**L14**	Burns *et al.* review[S2]	**L3.51**
**L15**	Hongyi Li Thesis[S4]	**27b**
**L16**	Hongyi Li Thesis[S4]	**28b**
**L17**	Hongyi Li Thesis[S4]	**29b**
**L18**	This paper	**L18**
**L19**	This paper	**L19**
**L20**	Rit *et al.*[S3]	**8a**
**L21**	Rit *et al.*[S3]	**10a**

Methods S1: data augmentation strategy, relates to Figure 2.

A data augmentation strategy was applied to balance the dataset. We duplicated the dataset size. As given chiral ligand produces a specific enantiomer, then the enantiomer of the chiral ligand must produce the enantiomer of the first product. This way, we have taken all the reactions in the original Owen *et al.* database, and we have created a second reaction, which shares the exact same conditions as the original reaction, with only two changes:

1. The chiral ligand SMILES was changed to the SMILES of its enantiomer.
2. The %top variable was rewritten to represent the change in chirality of the product by calculating the subtraction 100-%top.

With these two slight changes, we were able to double the size of the original database and to balance the dataset (Figure S11).

In terms of the reaction representation, for the GNN, the change above didn’t imply any new considerations nor changes, as our script automatically takes the SMILES and creates the representation of the molecules. Since these new reactions already had the correct SMILES of the enantiomer of the original chiral ligand, then the representation created does correspond to the intended “new” ligand. For the case of the Owen *et al.* database[S1] (Reference 20 in the main paper), we had to make an extra modification. Since Owen’s approach implicitly encodes the chirality of the ligand by assigning indexes to the carbons of the 1,3-cyclohexadiene structure, then the change of

chirality must consider that the numeration of carbons also does change. Therefore, these new "virtual" ligands have been numerated considering the correct numeration rules created by Owen *et al.*[S1] to further calculate the features of each diene substituent (R^n, n = 1-6) and allocate such information correctly in the database. This process is equivalent to exchange the descriptors of the "real" and "virtual" ligand between positions 1 and 3, and 4 and 6 (shown in Figures S3 and S6).

Methods S2: preparation and use of 'unseen' ligands, relates to Figure 2.

In the process of attaining our best Himbert diene ligands for RhCAA chemistry (Ref. 45 in the main paper) we prepared a significant number of development ligands (see Table S4). These ligands, which HCat-GNet had never been trained on, constitute an ideal test set in 'unseen' but nearby chemical space. Details of the preparation of these development ligands are given below.

Reactions, reagents and solvents. All air-sensitive reactions were carried out under an inert atmosphere using oven-dried apparatus. All commercially available reagents were used as received unless otherwise stated. Petroleum ether refers to Sigma-Aldrich product 24587 (petroleum ether boiling point 40-60 °C).

Chromatography. Thin layer chromatography (TLC) was performed on Merck DF-Alufoilien $60F_{254}$ 0.2 mm precoated plates. Compounds were visualized by exposure to UV light or by dipping the plates into solutions of potassium permanganate followed by gentle heating.

Melting Points. Melting points were recorded on a Gallenkamp melting point apparatus and are uncorrected. The solvent of recrystallization is reported in parentheses.

Spectroscopic data. Infrared (IR) spectra were recorded on Bruker platinum alpha FTIR spectrometer on the neat compound using the attenuated total refraction technique. Proton and ^{13}C NMR spectra were referenced to external tetramethylsilane via the residual protonated solvent (^1H) or the solvent itself (^{13}C). All chemical shifts are reported in parts per million (ppm). For $CDCl_3$, the shifts are referenced to 7.26 ppm for ^1H NMR spectroscopy and 77.16 ppm for ^{13}C NMR spectroscopy. Coupling constants (*J*) are quoted to the nearest 0.1 Hz. Electrospray ionisation (ESI) high-resolution mass spectrometry (HRMS) analyses were performed on a Bruker micrOTOFII mass spectrometer (Bruker Daltonik, Bremen, Germany), interfaced to an Agilent 1200 HPLC (Agilent Technologies, Santa Clara, USA). Samples were presented in solution for analysis by Flow Injection, 1 µL of solution being injected into the ion source of the instrument along with a flow of 0.2 mL min^{-1} of 70% $MeOH/H_2O$ eluent. The mass spectrometer was operated in electrospray ionisation (ESI) mode at a typical resolving power of 8000. Control of the analysis was performed through Bruker's Compass Open Access QC automated data acquisition and reporting software (v1.3; Bruker Daltonik, Bremen, Germany).

Enantioselective additions of phenyl boronic acid to 2-cyclohexen-1-one

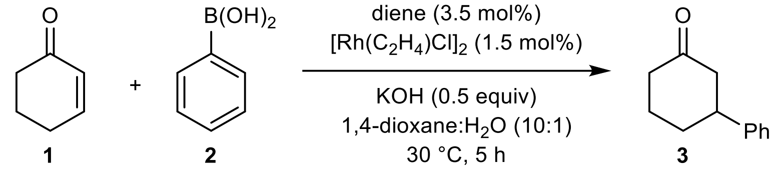

A solution of $[Rh(C_2H_4)_2Cl]_2$ (1.5 mol%) and chiral diene (3.5 mol%) in 1,4-dioxane was stirred at room temperature for 15 min. Phenylboronic acid (**2**) (2.0 equiv) and KOH (0.5 equiv) followed by 1,4-dioxane and H_2O were added. Finally, 2-cyclohexen-1-one (**1**) (0.30 mmol or 0.10 mmol) was added and the mixture was heated at 30 °C for 5 h. The reaction was cooled to room temperature and the solvent was removed *in vacuo*. The crude residue was purified by column chromatography (0-15% EtOAc/petroleum ether) to give 3-phenylcyclohexan-1-one (**3**) as a colourless oil. R_f = 0.43 (10% EtOAc/petroleum ether); IR 3027, 2934, 1709, 1495, 1450, 1222, 1029, 754, 699, 538 cm^{-1}; ^1H NMR (400 MHz, $CDCl_3$) δ 7.33 (2H, t, *J* = 7.5 Hz), 7.28–7.19 (3H, m), 3.07–2.96 (1H, m), 2.65–2.51 (2H,

m), 2.51–2.30 (2H, m), 2.20–2.12 (1H, m), 2.12–2.04 (1H, m), 1.93–1.70 (2H, m); ^{13}C NMR (101 MHz, $CDCl_3$) δ 211.1, 144.5, 128.8, 126.8, 126.7, 49.0, 44.8, 41.3, 32.9, 25.6; HRMS (ESI) Exact mass calculated for $[C_{12}H_{15}O]^+$ $[M+H]^+$: 175.1117, found 175.1111. The spectroscopic data are consistent with those reported previously.

(R)-3-Phenylcyclohexan-1-one. Enantiomeric excess was determined by HPLC using a Chiralpak AS-H column (95:5 isohexane:*i*-PrOH, 1.0 mL/min, 210 nm, 25 °C); t_r (minor) = 17.4 min, t_r (major) = 19.3 min.
(S)-3-Phenylcyclohexan-1-one. Enantiomeric excess was determined by HPLC using a Chiralpak AS-H column (95:5 isohexane:*i*-PrOH, 1.0 mL/min, 210 nm, 25 °C); t_r (major) = 16.2 min, t_r (minor) = 18.5 min.

Preparation of Himbert dienes. The synthetic fragments below were used.

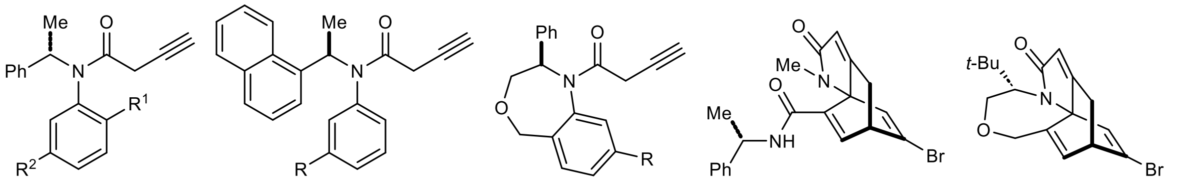

4a R^1 = Ph, R^2 = H
4b R^1 = H, R^2 = *o*-Tolyl
4c R^1 = Ph, R^2 = Ph

4d R = Ph
4e R = H

5a R = H
5b R = Me

6

7

General Procedure A. To a microwave tube was added alkyne **4a-5b** (1 equiv) and K_2CO_3 (0.1-0.5 equiv). The tube was sealed and then evacuated and backfilled three times with argon. Toluene was added and the mixture was heated at 145 °C for 18 h. The mixture was cooled to room temperature and then washed with H_2O and brine, dried (Na_2SO_4), filtered and concentrated *in vacuo*. Purification by silica gel chromatography or preparative TLC gave the desired products.

General Procedure B. A solution of (5*S*,7a*S*)-9-bromo-1-methyl-2-oxo-*N*-((*S*)-1-phenylethyl)-1,2,4,5-tetrahydro-5,7a-ethenoindole-7-carboxamide (**6**) (1.0 equiv), arylboronic acid (2.0 equiv), Pd(dppf)Cl_2 (2.5 mol%), K_2CO_3 (2.0 equiv) and toluene was heated at 110 °C for 8 h. The reaction was cooled to room temperature, H_2O was added, and the mixture was extracted with EtOAc (three times). The combined organic layers were dried (Na_2SO_4), filtered, and concentrated *in vacuo*. Purification of the residue by preparative TLC or column chromatography gave the arylation product.

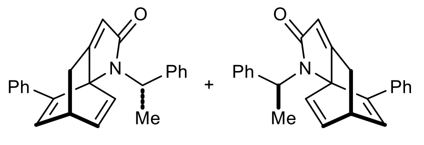

L1a **L1b**

*(5R,7aS)-7-Phenyl-1-((S)-1-phenylethyl)-4,5-dihydro-5,7a-ethenoindol-2(1H)-one (**L1a**) and (5S,7aR)-7-phenyl-1-((S)-1-phenylethyl)-4,5-dihydro-5,7a-ethenoindol-2(1H)-one (**L1b**).* The title compounds were prepared according to General Procedure A, with (*S*)-*N*-([1,1'-biphenyl]-2-yl)-*N*-(1-phenylethyl)but-3-ynamide (**4a**) (88 mg, 0.26 mmol) and K_2CO_3 (10 mg, 0.07 mmol) in toluene (3 mL). The crude mixture was passed through a short silica gel column with EtOAc, concentrated and then purified by preparative TLC (silica gel, 10% EtOAc/petroleum ether, 5 rounds) to give the desired products as pale-yellow solids (**L1a** = 15.3 mg, 17% yield; **L1b** = 16.2 mg, 18% yield). IR 3032, 1666, 1388, 1364, 1002, 834, 750, 716, 668, 576 cm^{-1}; HRMS (ESI) Exact mass calculated for $[C_{48}H_{42}N_2NaO_2]^+$ $[2M+Na]^+$: 701.3138, found: 701.3124.

Data for **L1a**: R_f = 0.22 (15% EtOAc/cyclohexane); m.p. 146-148 ℃ (EtOAc); $[\alpha]_D^{25}$ -20.0 (*c* 1.00, $CHCl_3$); ^{1}H NMR (400 MHz, $CDCl_3$) δ 7.43-7.27 (6H, m), 7.25-7.05 (4H, m), 6.37 (1H, dd, *J* = 7.5, 6.1 Hz), 6.27 (1H, d, *J* = 6.2 Hz), 5.96 (1H, dd, *J* = 7.5, 1.6 Hz), 5.88 (1H, t, *J* = 1.7 Hz), 4.18 (1H, q, *J* = 7.3 Hz), 4.08-3.97 (1H, m), 2.52-2.27 (2H, m), 1.43 (3H, d, *J* = 7.2 Hz); ^{13}C NMR (126 MHz, $CDCl_3$) δ 175.1, 160.9, 144.6, 143.8, 137.4, 134.4, 132.2, 130.9, 128.6, 128.2, 127.8, 127.6, 127.0, 126.9, 116.7, 58.1, 38.6, 31.1, 20.9.
Data for **L1b**: R_f = 0.18 (15% EtOAc/cyclohexane); m.p. 126-128 ℃ (EtOAc); $[\alpha]_D^{25}$ +60.0 (*c* 1.04, $CHCl_3$); ^{1}H NMR (500 MHz, $CDCl_3$) δ7.23-7.11 (8H, m), 7.00-6.94 (2H, m), 6.49 (1H, dd, *J* = 7.5, 6.2 Hz), 6.35 (1H, dd, *J* = 7.6, 1.7 Hz), 6.32 (1H, d, *J* = 6.3 Hz), 5.94 (1H, t, *J* = 1.8 Hz), 5.06 (1H, q, *J* =

7.4 Hz), 4.09 (1H, m), 2.48 (1H, m), 2.38 (1H, m), 1.23 (3H, d, *J* = 7.4 Hz); ^{13}C NMR (126 MHz, CDCl3): 174.9, 162.4, 144.1, 143.8, 138.2, 133.8, 132.3, 131.0, 128.3, 128.3, 127.2, 126.9, 126.6, 115.7, 53.9, 38.5, 30.9, 19.8.

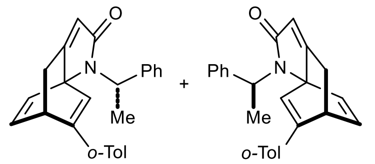

L2a **L2b**

*(5S,7aS)-1-((S)-1-Phenylethyl)-6-(o-tolyl)-4,5-dihydro-5,7a-ethenoindol-2(1H)-one (**L2a**) and (5R,7aR)-1-((S)-1-phenylethyl)-6-(o-tolyl)-4,5-dihydro-5,7a-ethenoindol-2(1H)-one (**L2b**).* The title compounds were prepared according to General Procedure A, with (*S*)-*N*-(2'-methyl-[1,1'-biphenyl]-3-yl)-*N*-(1-phenylethyl)but-3-ynamide (**4b**) (424.2 mg, 1.2 mmol) and K_2CO_3 (40 mg, 0.29 mmol) in toluene (9 ml). The crude mixture was passed through a short silica gel column with EtOAc, concentrated and then purified by column chromatography (silica gel, 10-30% EtOAc in cyclohexane) to give the desired products as yellow oils (**L2a** = 90.9 mg, 21% yield; **L2b** = 30.4 mg, 7% yield). IR 3059, 1675, 1491, 1323, 1267, 834, 755, 722, 675, 564 cm^{-1}; HRMS (ESI) Exact mass calculated for $[C_{25}H_{24}NO]^+$ $[M+H]^+$: 354.1852, found: 354.1851.

Data for **L2a**: R_f = 0.24 (15% EtOAc/cyclohexane); m.p. 83-85 ℃ (Et_2O); $[\alpha]_D^{25}$ +108 (*c* 1.00, $CHCl_3$); ^{1}H NMR (400 MHz, $CDCl_3$) δ 7.50 (2H, d, *J* = 7.5 Hz), 7.37-7.27 (3H, m), 7.22-7.12 (3H, m), 6.93 (1H, dd, *J* = 7.2 Hz), 6.51 (1H, dd, *J* = 7.4, 6.1 Hz), 6.44 (1H, dd, *J* = 7.5, 1.6 Hz), 5.86 (1H, t, *J* = 1.8 Hz), 5.61 (1H, q, *J* = 7.3 Hz), 5.53 (1H, d, *J* = 2.0 Hz), 4.07 (1H, m), 2.38 (2H, dd, *J* = 16.6, 2.2 Hz), 2.06 (3H, s), 1.90 (3H, d, *J* = 7.3 Hz); ^{13}C NMR (101 MHz, $CDCl_3$): 173.9, 160.6, 145.8, 142.0, 133.5, 132.8, 130.5, 128.6, 127.7, 127.5, 127.2, 125.8, 115.4, 75.6, 51.9, 44.4, 30.5, 20.7, 19.3.
Data for **L2b**: R_f = 0.21 (15% EtOAc/cyclohexane); m.p. 73-75 ℃ (Et_2O); $[\alpha]_D^{25}$ -40 (*c* 1.00, $CHCl_3$); ^{1}H NMR ($CDCl_3$, 500 MHz) δ 7.54–7.41 (2H, m), 7.39–7.20 (2H, m), 7.20– 7.07 (3H, m), 6.94 (1H, d, *J* = 7.2 Hz), 6.51 (1H, dd, *J* = 7.5, 6.1 Hz), 6.44 (1H, dd, *J* = 7.4, 1.6 Hz), 5.86 (1H, t, *J* = 1.7 Hz), 5.61 (1H, q, *J* = 7.3 Hz), 5.53 (1H, d, *J* = 2.0 Hz), 4.07 (1H, m), 2.48–2.23 (2H, m), 2.06 (3H, s), 1.90 (3H, d, *J* = 7.5 Hz); ^{13}C NMR (101 MHz, $CDCl_3$): 173.9, 160.6, 145.8, 142.0, 133.5, 132.8, 130.5, 128.6, 127.7, 127.5, 127.2, 125.8, 115.4, 75.6, 51.9, 44.4, 30.5, 20.7, 19.3.

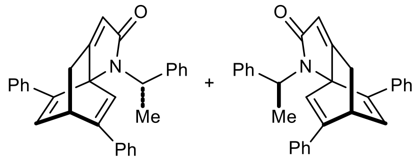

L3a **L3b**

*(5S,7aS)-6,8-Diphenyl-1-((S)-1-phenylethyl)-4,5-dihydro-5,7a-ethenoindol-2(1H)-one (**L3a**) and (5R,7aR)-6,8-diphenyl-1-((S)-1-phenylethyl)-4,5-dihydro-5,7a-ethenoindol-2(1H)-one (**L3b**).* The title compounds were prepared according to General Procedure A, with (*S*)-*N*-([1,1':4',1''-terphenyl]-2'-yl)-*N*-(1-phenylethyl)but-3-ynamide (**4c**) (210 mg, 0.42 mmol) and K_2CO_3 (20 mg, 0.15 mmol) and toluene (9 ml). The crude mixture was passed through a short silica gel column with EtOAc, concentrated and then purified by preparative TLC (silica gel, 5% EtOAc in petroleum ether, 4 rounds) to give the desired products as pale-yellow solids (<15 mg for each isomer).

Characterization was performed on a mixture of isomers **L3a** and **L3b**. IR 2922, 1673, 1490, 1443, 1326, 1021, 835, 751, 695, 563 cm^{-1}; ^{1}H NMR (500 MHz, $CDCl_3$) δ 7.45-7.42 (2H, m), 7.39-7.26 (14H, m), 7.25-7.17 (10H, m), 7.14-7.09 (2H, m), 7.02-6.99 (2H, m), 6.46-6.39 (3H, m), 6.08 (1H, d, *J* = 2.2 Hz), 5.96 (2H, dt, *J* = 29.3, 1.7 Hz), 5.13 (1H, q, *J* = 7.4 Hz), 4.57-4.46 (2H, m), 4.23 (1H, q, *J* = 7.1 Hz), 2.57-2.43 (4H, m), 1.48 (3H, d, *J* = 7.2 Hz), 1.27 (3H, d, *J* = 7.2 Hz); ^{13}C NMR (126 MHz, $CDCl_3$): 175.1, 174.9, 162.6, 161.0, 146.1, 145.4, 144.7, 144.6, 144.5, 143.7, 138.1, 137.2, 136.2, 136.1, 132.2, 132.0, 128.8, 128.7, 128.6, 128.4, 128.3, 128.3, 128.1, 128.0, 127.7, 127.7, 127.4, 127.1, 127.0, 126.9, 126.7, 125.1, 124.9, 124.7, 124.5, 116.7, 115.7, 80.2, 78.5, 58.2, 54.0, 40.9, 40.8, 31.2, 31.0, 20.8, 20.0; HRMS (ESI) Exact mass calculated for $[C_{30}H_{26}NO]^+$ $[M+H]^+$: 416.2009, found: 416.2006.

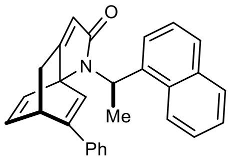
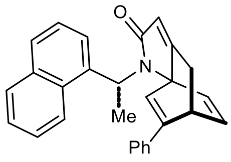

L4a **L4b**

*(5S,7aS)-1-((R)-1-(Naphthalen-1-yl)ethyl)-6-phenyl-4,5-dihydro-5,7a-ethenoindol-2(1H)-one (**L4a**) and (5R,7aR)-1-((R)-1-(naphthalen-1-yl)ethyl)-6-phenyl-4,5-dihydro-5,7a-ethenoindol-2(1H)-one (**L4b**).* The title compounds were prepared according to General Procedure A, with (*R*)-*N*-([1,1'-biphenyl]-3-yl)-*N*-(1-(naphthalen-1-yl)ethyl)but-3-ynamide (**4d**) (200 mg, 0.5 mmol), K_2CO_3 (40.0 mg, 0.25 mmol) and toluene (2 mL). The crude mixture was purified by column chromatography (20% EtOAc in cyclohexane) to give the desired products as pale-yellow solids (**L4a** = 82.4 mg, 41% yield, **L4b =** 80.8 mg, 40% yield). IR 2920, 1663, 1597, 1348, 1300, 959, 808, 770, 629, 550 cm^{-1}; HRMS (ESI) Exact mass calculated for $[C_{28}H_{24}NO]^+$ $[M+H]^+$: 390.1852, found: 390.1844.

Data for **L4a**: R_f = 0.19 (20% EtOAc/cyclohexane); m.p. 72-74 °C (Et_2O); $[\alpha]_D^{25}$ -176 (*c* 1.00, $CHCl_3$); ^{1}H NMR (400 MHz, $CDCl_3$) δ 8.27 (1H, d, *J* = 8.5 Hz), 7.87 (1H, d, *J* = 9.6 Hz), 7.82 (1H, d, *J* = 8.2 Hz), 7.72 (1H, d, *J* = 7.1 Hz), 7.58 (1H, ddd, *J* = 8.5, 6.8, 1.5 Hz), 7.51 (1H, ddd, *J* = 8.0, 6.8, 1.2 Hz), 7.48-7.41 (1H, m), 7.35 (3H, d, *J* = 4.3 Hz), 7.33-7.26 (1H, m), 6.63-6.52 (2H, m), 5.96 (1H, dd, *J* = 7.5, 6.2 Hz), 5.89 (1H, t, *J* = 1.8 Hz), 4.65 (1H, dd, *J* = 7.4, 1.5 Hz), 4.27 (1H, dq, *J* = 6.5, 2.2 Hz), 2.36-2.14 (3H, m), 2.03 (3H, d, *J* = 7.2 Hz); ^{13}C NMR (101 MHz, $CDCl_3$) δ 173.5, 161.0, 145.6, 138.4, 135.7, 133.6, 132.0, 131.8, 131.7, 131.5, 128.8, 128.7, 128.0, 127.1, 126.0, 125.2, 125.2, 124.7, 124.7, 123.5, 114.8, 46.8, 41.0, 30.2, 29.7, 19.4.

Data for **L4b**: R_f = 0.13 (20% EtOAc/cyclohexane); m.p. 172-174 °C (Et_2O); $[\alpha]_D^{25}$ +8 (*c* 1.00, $CHCl_3$); ^{1}H NMR (400 MHz, $CDCl_3$) δ 8.28 (1H, d, *J* = 8.5 Hz), 7.91 (1H, d, *J* = 6.5 Hz), 7.88 (1H, d, *J* = 8.2 Hz), 7.74 (1H, d, *J* = 7.1 Hz), 7.62-7.34 (4H, m), 7.14 (2H, dd, *J* = 5.9, 1.7 Hz), 6.73 (2H, dd, *J* = 7.5, 2.1 Hz), 6.66-6.52 (2H, m), 6.47-6.39 (1H, t, *J* = 7.2 Hz), 5.88-5.80 (1H, m), 4.61 (1H, d, *J* = 2.1 Hz), 4.26 (1H, dt, *J* = 6.5, 2.0 Hz), 2.24 (2H, qt, *J* = 16.5, 2.2 Hz), 2.01 (3H, d, *J* = 7.2 Hz); ^{13}C NMR (101 MHz, $CDCl_3$) δ 173.3, 162.8, 160.7, 153.7, 143.3, 135.8, 133.7, 133.4, 132.1, 131.7, 128.8, 128.7, 128.4, 127.5, 127.3, 127.2, 126.1, 125.6, 125.3, 124.7, 124.5, 123.6, 114.8, 46.7, 40.5, 30.3, 19.2.

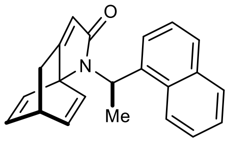

L5

*(5R,7aR)-1-((R)-1-(Naphthalen-1-yl)ethyl)-4,5-dihydro-5,7a-ethenoindol-2(1H)-one (**L5**).* The title compound was prepared according to General Procedure A, with (*R*)-*N*-(1-(naphthalen-1-yl)ethyl)-*N*-phenylbut-3-ynamide (**4e**) (313.3 mg, 1 mmol), K_2CO_3 (30 mg, 0.22 mmol) and toluene (9 mL). The crude mixture was passed through a short silica gel column with EtOAc, concentrated and then purified by preparative TLC (silica gel, 30% EtOAc/cyclohexane) to give the desired product as a yellow solid (222.7 mg, 71% yield). R_f = 0.49 (30% EtOAC/cyclohexane); m.p. 162-164 ℃ (EtOAc); $[\alpha]_D^{25}$ -20 (*c* 1.00, $CHCl_3$); IR 2928, 1737, 1385, 1357, 1262, 869, 827, 708, 539, 411 cm^{-1}; ^{1}H NMR (400 MHz, $CDCl_3$) δ 8.24 (1H, d, *J* = 8.5 Hz), 7.86 (2H, m), 7.81 (1H, d, *J* = 8.2 Hz), 7.78 (1H, d, *J* = 7.2 Hz), 7.61-7.38 (3H, m), 6.59-6.46 (2H, m), 6.40-6.28 (1H, m), 5.88-5.71 (2H, m), 4.55 (1H, d, *J* = 7.5), 387-3.79 (1H, m), 2.19-2.02 (2H, m), 1.97 (3H, d, *J* = 7.2 Hz); ^{13}C NMR (101 MHz, $CDCl_3$): 173.3, 160.8, 135.8, 133.6, 133.4, 132.1, 131.8, 131.1, 131.0, 128.8, 128.7, 127.1, 126.0, 125.2, 124.6, 123.5, 114.8, 75.1, 46.7, 38.4, 30.1, 19.3; HRMS (ESI) Exact mass calculated for $[C_{22}H_{20}NO]^+$ $[M+H]^+$: 314.1539, found: 314.1539.

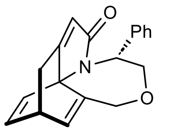

L7

*(5R,10S,12aR)-5-Phenyl-5,6,8,10-tetrahydro-3H-1,10-methanobenzo[e]pyrrolo[1,2-d][1,4]oxazepin-3-one (**L7**).* The title compound was prepared according to General Procedure A, with (*R*)-1-(2-phenyl-2,3-dihydrobenzo[*e*][1,4]oxazepin-1(5*H*)-yl)but-3-yn-1-one (**5a**) (116.5 mg, 0.4 mmol), K_2CO_3 (10 mg, 0.07 mmol) and toluene (2 mL). The crude mixture was passed through a short silica gel column with EtOAc, concentrated and then purified by preparative TLC (silica gel, 30% EtOAc/cyclohexane for 3 rounds) to give the desired product as a yellow solid (83.4 mg, 72% yield). R_f = 0.13 (30% EtOAc/cyclohexane); m.p. 127-129 ℃ (EtOAc); $[\alpha]_D^{25}$ -36 (*c* 1.00, $CHCl_3$); IR 2947, 1673, 1494, 1312, 1105, 982, 806, 696, 631, 584 cm^{-1}; ^{1}H NMR (400 MHz, $CDCl_3$) δ 7.73-7.60 (2H, m), 7.40-7.26 (3H, m), 6.21-6.01 (2H, m), 5.87-5.77 (2H, m), 5.04 (1H, dd, *J* = 7.5, 1.6 Hz), 4.62 (1H, dd, *J* = 13.0, 1.6 Hz), 4.50 (1H, d, *J* = 14.3 Hz), 4.03-3.92 (2H, m), 3.91-3.83 (1H, m), 2.22-2.14 (2H, m); ^{13}C NMR (101 MHz, $CDCl_3$): 173.3, 161.4, 142.8, 138.6, 132.3, 131.3, 129.2, 128.6, 128.0, 127.9, 114.5, 76.4, 76.3, 71.7, 54.8, 38.0, 30.3; HRMS (ESI) Exact mass calculated for $[C_{19}H_{18}NO_2]^+$ $[M+H]^+$: 292.1338, found: 292.1342.

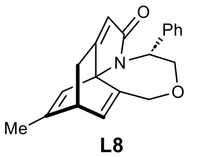

L8

*(5R,10R,12aR)-11-Methyl-5-phenyl-5,6,8,10-tetrahydro-3H-1,10-methanobenzo[e]pyrrolo[1,2-d][1,4]oxazepin-3-one (**L8**).* The title compound was prepared according to General Procedure A, with (*R*)-1-(8-methyl-2-phenyl-2,3-dihydrobenzo[*e*][1,4]oxazepin-1(5*H*)-yl)but-3-yn-1-one (**5b**) (152.7 mg, 0.50 mmol), K_2CO_3 (15 mg, 0.11 mmol) and toluene (2 mL). The crude mixture was passed through a short silica gel column with EtOAc, concentrated and then purified by column chromatography (35% EtOAc/cyclohexane) to give the desired product as a pale yellow solid (127.2 mg, 83% yield). R_f = 0.19 (35% EtOAc/cyclohexane); m.p. 162-164 ℃ (EtOAc); $[\alpha]_D^{25}$ -20 (*c* 1.00, $CHCl_3$); IR: 2928, 1737, 1385, 1357, 1262, 869, 827, 708, 539, 411 cm^{-1}; ^1H NMR (400 MHz, $CDCl_3$) δ 7.68-7.61 (2H, d, *J* = 7.1 Hz), 7.36-7.28 (3H, m), 6.15 (1H, dd, *J* = 6.1, 1.9 Hz), 5.84-5.76 (2H, m), 4.66-4.56 (2H, m), 4.48 (1H, d, *J* = 14.2 Hz), 4.03-3.93 (2H, m), 3.59- 3.50 (1H, m), 2.22-2.13 (2H, m), 1.62 (3H, d, *J* = 1.6 Hz); ^{13}C NMR (101 MHz, $CDCl_3$) δ 173.3, 161.4, 142.8, 138.6, 132.3, 131.3, 129.2, 128.6, 128.0, 127.9, 114.5, 76.4, 76.3, 71.7, 54.8, 38.0, 30.3; HRMS (ESI) Exact mass calculated for $[C_{20}H_{20}NO_2]^+$ $[M+H]^+$: 306.1494, found: 306.1496.

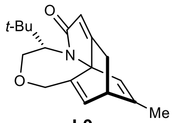

L9

*(5S,10S,12aS)-5-(tert-Butyl)-11-methyl-5,6,8,10-tetrahydro-3H-1,10-methanobenzo[e]pyrrolo[1,2-d][1,4]oxazepin-3-one (**L9**).* The title compound was prepared according to General Procedure A, with (*S*)-1-(2-(*tert*-butyl)-8-methyl-2,3-dihydrobenzo[*e*][1,4]oxazepin-1(5*H*)-yl)but-3-yn-1-one (**7**) (57 mg, 0.20 mmol), K_2CO_3 (14 mg, 0.10 mmol) and toluene (1 mL). R_f = 0.10 (50% EtOAc/pentane); $[\alpha]_D^{25}$-96 (*c* 1.00, $CHCl_3$); IR: 2960, 2873, 1682, 1443, 1366, 1319 1272, 1227, 1190, 1147 cm^{-1}; ^1H NMR (500 MHz, $CDCl_3$) δ 6.19 (1H, dd, *J* = 6.1, 2.0 Hz), 6.09-6.07 (1H, m), 5.79 (1H, t, *J* = 1.7 Hz), 4.30 (1H, dd, *J* = 6.5, 3.0 Hz), 4.26 (1H, d, *J* = 13.8 Hz), 4.08 (1H, dd, *J* = 13.4, 6.6 Hz), 3.92 (1H, ddd, *J* = 13.8, 2.1, 0.8 Hz), 3.76 (1H, dd, *J* = 13.4, 3.1 Hz), 3.63 (1H, app dq, *J* = 6.2, 2.4 Hz), 2.27–2.21 (1H, m), 2.18 (1H, dt, *J* = 16.4, 2.2 Hz), 1.91 (3H, d, *J* = 1.7 Hz), 1.11 (9H, s); ^{13}C NMR (126 MHz, $CDCl_3$) δ 176.2, 162.0, 142.8, 142.6, 128.7, 123.7, 114.6, 77.1, 69.5, 68.0, 62.3, 43.6, 35.3, 30.1, 28.5, 19.7; HRMS (ESI) Exact mass calculated for $[C_{18}H_{23}NNaO_2]^+$ $[M+Na]^+$: 308.1621, found: 308.1426.

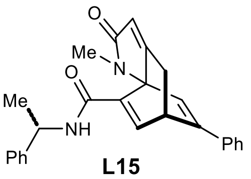

L15

*(5S,7aS)-1-Methyl-2-oxo-9-phenyl-N-((S)-1-phenylethyl)-1,2,4,5-tetrahydro-5,7a-ethenoindole-7-carboxamide (**L15**).* The title compound was prepared according to General Procedure B with using (5*S*,7a*S*)-9-bromo-1-methyl-2-oxo-*N*-((*S*)-1-phenylethyl)-1,2,4,5-tetrahydro-5,7a-ethenoindole-7-carboxamide (**6**) (39.9 mg, 0.1 mmol), phenylboronic acid (24.4 mg, 0.2 mmol) Pd(dppf)Cl_2 (1.9 mg, 2.5 μmol), K_2CO_3 (27.4 mg, 0.2 mmol) and toluene (1.0 mL). The crude mixture was purified by preparative TLC (80% EtOAc/cyclohexane) to give the desired product as a pale-yellow solid (30.3 mg, 82% yield). R_f = 0.32 (80% EtOAc/cyclohexane); $[\alpha]_D^{25}$+180 (*c* 0.20, $CHCl_3$); m.p. 244-246 °C (Et_2O); ^1H NMR (400 MHz, $CDCl_3$) δ 7.48–7.28 (8H, m), 7.20 (1H, d, *J* = 6.1 Hz), 6.31 (1H, d, *J* = 2.0 Hz), 6.06 (1H, d, *J* = 7.9 Hz), 5.92 (1H, d, *J* = 1.8 Hz), 5.18 (1H, p, *J* = 7.0 Hz), 4.54 (1H, dd, *J* = 6.6, 2.3 Hz), 2.98 (3H, s), 2.53 (2H, m), 1.49 (3H, d, *J* = 6.8 Hz); ^{13}C NMR (101 MHz, $CDCl_3$) δ 174.5, 160.8, 151.7, 146.6, 139.7, 139.8, 129.3, 128.9, 128.7, 128.5, 127.7, 126.3, 125.2, 122.8, 119.4, 115.5, 49.5, 41.8, 30.1, 28.3, 21.6; HRMS (ESI) Exact mass calculated for $[C_{26}H_{24}N_2NaO]^+$ $[M+Na]^+$: 419.1730, found: 419.1720.

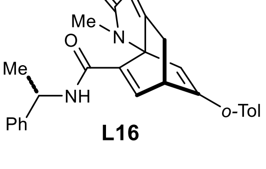

L16

*(5S,7aS)-1-Methyl-2-oxo-N-((S)-1-phenylethyl)-9-(o-tolyl)-1,2,4,5-tetrahydro-5,7a-ethenoindole-7-carboxamide (**L16**).* The title compound was prepared according to General Procedure B with using (5*S*,7a*S*)-9-bromo-1-methyl-2-oxo-*N*-((*S*)-1-phenylethyl)-1,2,4,5-tetrahydro-5,7a-ethenoindole-7-carboxamide (**6**) (39.9 mg, 0.1 mmol), 2-tolylboronic acid (27.2 mg, 0.2 mmol), Pd(dppf)Cl_2 (1.9 mg, 2.5 μmol), K_2CO_3 (27.4 mg, 0.2 mmol) and toluene (1.0 mL). The crude mixture was purified by preparative TLC (80% EtOAc/cyclohexane) to give the desired product as a pale yellow solid (30.4 mg, 76% yield). R_f = 0.22 (90% EtOAc/cyclohexane); $[\alpha]_D^{25}$+60.0 (*c* 0.50, $CHCl_3$); m.p. 253-255 °C (Et_2O); IR 3279, 2974, 1649, 1521, 1493, 1422, 1136, 835, 756, 723, 542 cm^{-1}; ^1H NMR (400 MHz, $CDCl_3$) δ 7.44–7.25 (5H, m), 7.27–7.09 (3H, m), 6.99 (1H, d, *J* = 7.0 Hz), 6.13 (1H, d, *J* = 7.9 Hz), 6.07 (1H, d, *J* = 2.0 Hz), 5.95 (1H, d, *J* = 1.8 Hz), 5.17 (1H, p, *J* = 7.1 Hz), 4.21 (1H, dd, *J* = 6.6, 2.3 Hz), 2.95 (3H, m), 2.58 (1H, m), 2.46 (1H, m), 2.24 (3H, s), 1.49 (3H, d, *J* = 6.9 Hz); ^{13}C NMR (101 MHz, $CDCl_3$) δ 174.3, 162.5, 148.1,

142.6, 140.0, 138.6, 137.8, 137.3, 135.4, 130.7, 128.9, 128.2, 128.1, 127.8, 126.3, 126.0, 125.1, 115.4, 49.2, 44.5, 29.9, 28.3, 21.6, 20.6; HRMS (ESI) Exact mass calculated for $[C_{27}H_{27}N_2O_2]^+$ $[M+H]^+$: 411.2067, found: 411.2065.

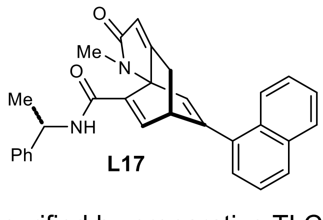

L17

*(5S,7aS)-1-Methyl-9-(naphthalen-1-yl)-2-oxo-N-((S)-1-phenylethyl)-1,2,4,5-tetrahydro-5,7a-ethenoindole-7-carboxamide (**L17**).* The title compound was prepared according to General Procedure B with using (5*S*,7a*S*)-9-bromo-1-methyl-2-oxo-*N*-((*S*)-1-phenylethyl)-1,2,4,5-tetrahydro-5,7a-ethenoindole-7-carboxamide (**6**) (39.9 mg, 0.1 mmol), 1-naphthalen boronic acid (34.4 mg, 0.2 mmol), Pd(dppf)Cl_2 (1.9 mg, 2.5 µmol), K_2CO_3 (27.4 mg, 0.2 mmol) and toluene (1.0 mL). The crude was purified by preparative TLC (80% EtOAc/cyclohexane) to give the desired product as a pale yellow solid (40.9 mg, 92% yield). R_f = 0.21 (90% EtOAc/cyclohexane); $[\alpha]_D^{25}$ +24 (*c* 1.0, $CHCl_3$); m.p. 196-198 °C (Et_2O); IR 3305, 2924, 1736, 1673, 1594, 1420, 827, 799, 703, 550 cm^{-1}; ^1H NMR (400 MHz, $CDCl_3$) δ 7.97–7.79 (2H, m), 7.74–7.63 (1H, m), 7.57–7.27 (7H, m), 7.19 (1H, dd, *J* = 7.0, 1.2 Hz), 6.28 (1H, d, *J* = 1.9 Hz), 6.20 (1H, d, *J* = 7.9 Hz), 6.01 (1H, d, *J* = 1.9 Hz), 5.21 (1H, p, *J* = 7.1 Hz), 4.35 (1H, dd, *J* = 6.2, 2.6 Hz), 2.99 (3H, s), 2.65 (1H, m), 2.52 (1H, m), 1.52 (3H, d, *J* = 6.9 Hz); ^{13}C NMR (101 MHz, $CDCl_3$) δ 175.7, 167.9, 139.9, 135.5, 131.0, 129.0, 128.7, 128.7, 127.8, 126.6, 126.4, 126. 1 (CH) 126.0, 125.6, 125.3, 125.3, 125.0, 49.3, 45.4, 29.89, 28.3, 21.3; HRMS (ESI) Exact mass calculated for $[C_{30}H_{27}N_2O_2]^+$ $[M+H]^+$: 447.2067, found: 447.2062.

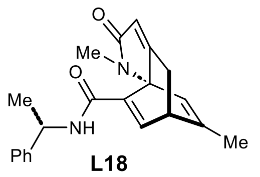

L18

*(5S,7aS)-1,9-Dimethyl-2-oxo-N-((S)-1-phenylethyl)-1,2,4,5-tetrahydro-5,7a-ethenoindole-7-carboxamide (**L18**).* An oven dried Schlenk tube was charged with XPhos (14.3 mg, 0.03 mmol) and $Pd_2(dba)_3$ (13.7 mg, 0.02 mmol). THF (4 mL), (5*S*,7a*S*)-9-bromo-1-methyl-2-oxo-*N*-((*S*)-1-phenylethyl)-1,2,4,5-tetrahydro-5,7a-ethenoindole-7-carboxamide (**6**) (200 mg, 0.50 mmol) and then a solution of DABAL-Me_3 (256 mg, 1.00 mmol) in THF (1 mL) were added. The mixture was heated at 66 °C for 4 h, cooled to room temperature and quenched carefully with 2 M aqueous HCl solution (2 mL). The aqueous phase was extracted with TBME and the combined organic phases were passed through a short plug of silica using TBME as eluent and then concentrated *in vacuo*. The crude material was purified by column chromatography to give the desired product. ^1H NMR (400 MHz, $CDCl_3$) δ 7.39-7.27 (5H, m), 7.10 (1H, d, *J* = 6.4 Hz), 6.05 (1H, d, *J* = 7.9 Hz), 5.85-5.81 (1H, m), 5.74 (1H, t, *J* = 1.6 Hz), 5.14 (1H, app p, *J* = 7.1 Hz), 3.79 (1H, app dq, *J* = 7.0, 2.4 Hz), 2.87 (3H, s), 2.40 (1H, app dt, *J* = 16.7, 2.2 Hz), 2.33 (1H, app dt, *J* = 16.7, 2.3 Hz), 1.87 (3H, d, *J* = 1.7 Hz), 1.46 (3H, d, *J* = 6.9 Hz); HRMS (ESI) Exact mass calculated for $[C_{21}H_{23}N_2O_2]^+$ $[M+H]^+$: 335.1754, found: 335.1753.

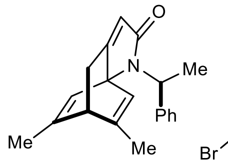

L6

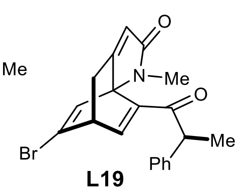

L19

Other ligands studies. Two additional development ligands **L6** and **L19** were prepared at micro-scale. Sufficient material was attained to allow their separation and trial in catalytic conjugate addition of $PhB(OH)_2$ to cyclohexenone. Due to modest performance or solubility issues they were not developed further.

Data for **L6**: R_f = 0.19 (30% EtOAc/pentane); $[\alpha]_D^{25}$ +28 (*c* 1.00, $CHCl_3$); IR: 2968, 2932, 2908, 1677, 1494, 1444, 1342, 823, 774, 700 cm^{-1}; ^1H NMR (400 MHz, $CDCl_3$) δ 7.49-7.43 (2H, m), 7.35-7.28 (2H, m), 7.28-7.22 (1H, m), 5.79-5.76 (1H, m), 5.72 (1H, t, *J* = 1.8 Hz), 5.47 (1H, q, *J* = 7.3 Hz), 5.24-5.20 (1H, m), 3.35 (1H, app. p, *J* = 2.4 Hz), 2.27-2.14 (2H, m), 1.85 (3H, d, *J* = 7.4 Hz), 1.84 (3H, d, *J* = 1.7 Hz), 1.74 (3H, d, *J* = 1.7 Hz); ^{13}C NMR (101 MHz, $CDCl_3$,) δ 174.1, 162.6, 143.2, 142.9, 142.6, 128.6, 127.5, 127.3, 124.9, 123.6, 114.0, 75.4, 52.1, 49.7, 29.6, 19.7, 19.4; HRMS (ESI) Exact mass calculated for $[C_{20}H_{21}NNaO]^+$ $[M+Na]^+$: 314.1515, found: 314.1517.

Data for **L19**: R_f = 0.20 (80% EtOAc/pentane); $[\alpha]_D^{25}$ +8 (*c* 1.00, $CHCl_3$); IR: 3277, 2975, 1675, 1648, 1586, 1526, 1424, 1376, 1253, 732, 699 cm^{-1}; ^1H NMR (500 MHz, $CDCl_3$) δ 7.37-7.21 (5H, m), 6.99 (1H, d, *J* = 6.5 Hz), 6.34 (1H, d, *J* = 2.3 Hz), 5.94-5.87 (2H, m), 5.12 (1H, app. p, *J* = 7.1 Hz), 4.10 (1H, dq, *J* = 7.2, 2.6 Hz), 3.17 (3H, s), 2.63 (1H, app. dt, *J* = 16.8, 2.2 Hz), 2.39 (1H, app. dt, *J* = 16.8, 2.4 Hz), 1.50 (3H, d, *J* = 6.9 Hz); ^{13}C NMR (101 MHz, $CDCl_3$) δ 173.7, 162.6, 158.9, 142.7, 138.6, 137.6, 129.0, 128.2, 127.8, 126.2, 124.8, 116.2, 76.5, 49.2, 48.6, 29.8, 28.3, 22.0; HRMS (ESI) Exact mass calculated for $[C_{20}H_{19}{}^{79}BrN_2NaO_2]^+$ $[M+Na]^+$: 421.0523, found: 421.0518.

Methods S3: analysis of problematic substrates, relates to Figures 6-7

Analysis of poorly predicted high enantioselective reactions (Figure 6). To understand drivers of wrong predictions, we investigated our training data and the chemical diversity they contained (Figure S15 and Table S1). Structures **S1** and **S2** (Figure S15) share closely related topology around the reactive carbon. In **S2**, the molecule has a benzyl group. We hypothesize an error occurred as the training set did not have enough exemplar reactions with similar substrates, which led both methods to predict wrongly the selectivity of the reaction. We found 46 reactions in our training set had a benzyl unit and a nitrogen atom in the substrate structure. All of these were converted with lower selectivity than other reactions. We hypothesize that this biased the model to think this combination of groups in the substrate drives the poor selectivity.
Similarly, for substrate **S4** we investigated the effect of the nitro group on the learning set. The values of $\Delta\Delta G^{\ddagger}$ follow a different distribution from that found in full set, having a major frequency of low selectivity (*er*) values. This biases both models to predict that substrates with this functional group will exhibit low stereoselectivity reactions. Lastly, for **S5** we investigated the presence of sulfur atoms in the training dataset. We found that no substrate contained this type of atom, which clearly led to the large error. Additionally, this reaction differs in nature to those in the training set as it is a 1,2-addition (as opposed to a 1,4-addition). The differences in the test data of Figure 15 and the training data would rationalise the errors obtained.

Analysis of machine-given rankings of stereoselectivity (Figure 7). We discuss here in further detail the results of Figure 7 whose primary analysis is in **Table S2**.
For **Group 1** (see Fig. 7 main paper), ligand **L2** provides the lowest selectivity (65% *ee*), while **L1** attains 94%, and **L3** 96%. The structural difference between **L1** and **L2** is subtle, challenging the algorithms to identify that **L2** has the lower selectivity. HCat-GNet ranks all three ligands correctly.
For the **Group 2**, the ligand **L6** is the least selective and **L4** the most selective. HCat-GNet successfully identifies **L4** as the most selective but confuses the relative selectivity of **L5** and **L6**. Gradient Boosting ranked the three ligands correctly. Both methods were able to identify **L5** and **L6** as least selective ligands delivering significantly lower *ee* predictions (ca. 22% and 46% *ee* for HCat-GNet and 28% and 27% *ee* for Gradient Boosting after converting from $\Delta\Delta G^{\ddagger}$).
Lastly in **Group 3** the R^1, R^2 and R^7 substituents were joint by bonds within the ligand. We noticed that the phenyl substituent in the new cycle in general leads to poorer performance compared to the *tert*-butyl substituent. We wondered if the ML models would be able to differentiate this even though the models were not specifically trained on such molecules. HCat-GNet successfully identifies that the *tert*-butyl group will lead to a ligand that will be more selective among the group of catalysts. However, we noticed that **L7** and **L8** were ranked inversely. In the case of Gradient Boosting, although it 'understood' that for this set of ligands less substituents lead to less selective processes, it could not predict that the *tert*-butyl variant of the ligand family would provide higher selectivity. For **Group** 3 only Gradient Boosting was able to identify **L7** as a poor performing ligand (ca. 78% *ee*), while both models for the rest of ligands predicted high selectivity (>90% *ee*).

Method S4: graph denoiser tool, relates to Figure 10

In the GNNExplainer rankings of Figure 10 it is noticeable that for 'atom chirality', 'atom aromaticity', and 'atom in ring', some nodes are not displayed at all. This happens as those nodes (atoms) have a value of '0' for those features, and are therefore, not of relevance for those atoms. In general, for 'atom identity' there are not big differences among nodes but for 'degree' and 'hybridization' features, HCat-GNet finds that carbons with double bonds are more important for the final decision. This is likely because the sp^2 hybridized atoms within the chiral ligand are the ones that share a bond with the bulky group that causes the orientation of the substrate that leads to the stereoselectivity of the reaction. We propose the GNN is learning this relationship between ligand structure and reaction stereoselectivity, as demonstrated in Figure 10. For the feature 'in ring', atoms within a phenyl group have more importance than those in the ligand core structure. This is consistent as the GNN learns that external ring-substituents within the chiral ligand lead to bulkier groups, in comparison with acyclic counterparts, thus providing higher selectivity. Of course, the ligand core structure is always a cycle and therefore constant across all predictions.
A highly useful feature of the explainability software within HCat-GNet is that it can easily be applied to *any* of the participant molecules for *any* reaction within the database. We propose this tool is particularly valuable for ligand design, as it analyses which node properties are the most important for

an atom in a given location within the ligand. In this way, it would be possible to create optimal *de novo* catalyst structure for a specific asymmetric catalytic reaction. As the model has been trained on 8 folds of the training set, the explanations derived contain information from all the datapoints, effectively condensing the high dimensional information obtained in the training process in a simple graphical plot of high interpretability and meaning for humans.
In Figure S16 the molecule that HCat-GNet finds most important reaction component for the final prediction is the ligand, which is also in agreement with Figure 10. It is interesting to note that the algorithm also finds the whole ligand structure relevant in delivering predictions, and the atoms are the less important are those that are directly attached to the core ligand structure. In the case of the substrate, unsurprisingly the model finds the carbons forming the unsaturated system most important. However, this does indicate that HCat-GNet effectively gathers information about the chemical environment near the reactive site and can get deductions from it. For example, it can deduce if there are bulky groups around the area, which is related to the degree of saturation of the atoms there, due to the steric effects on reaction selectivity. The oxygen from the electron withdrawing group is not found to be as important, consistent with only limited roles for Lewis acid coordination effects in the reaction. Lastly, for the organoboron reagent, the phenyl group (nucleophile) is found to be important, while the remaining -$B(OH)_2$ is not found as important for the prediction. This is not unexpected as most samples in the training data have the same Ar-$B(OH)_2$ structure, and therefore the algorithm learns that is more relevant to gather information from the ligand substituents as explainers of the difference of selectivity between reactions. Additionally, it is known mechanistically that no boron species is not present in the stereochemically defining transition state, consistent with Figure S16.
As a second validation experiment of the GNNExplainer software (see Figure S17), we took the same reaction and plotted the three participant molecules (ligand, substrate, and organoboron reagent). This provides insights into the regions of these key components that the algorithm finds particularly important in delivering an *ee* selectivity prediction.

Methods S5: generalization, relates to Figure 12

To show the generality of HCat-GNet it was also applied to three very different, but ligand controlled, catalytic processes. For the locations of the primary data see the Star Methods Key Resources Table for their Github locations.

BiAryl Dataset. The new BiAryl dataset consists of 484 reactions (53 ligands, 107 substrates, and 98 boron reagents), taken from Reference S2. With this dataset, we show the HCat-GNet tool can be applied to other systems with minimum changes on the code. The graph representation used for this dataset is the same as the one used for the RhCASA datasets, but with the difference that coding of the BiAryl axial chirality is the “ligand configuration” (instead of the planar chirality of the diene ligands). Results are shown in Figure S13. HCat-GNet attains a better RMSE performance and the results are also human-interpretable. However, no Wilcoxon statistical difference was found against Gradient Boosted fits of CircuS fingerprints. This dataset models the behaviour of the new reaction dataset:

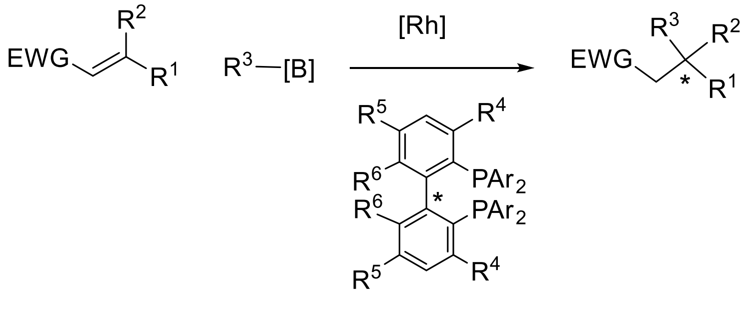

Catalytic Asymmetric DeAromatisation (CADA) Dataset. This dataset was taken directly from Gao *et al.*[S5] This dataset was curated by those authors encompassing a total of 847 reactions of CADA mediated by hypervalent iodine catalysts. This consists of two types of reactions: *ortho*-dearomatisation reactions (637 instances), and *para*-dearomatization reactions (210 instances). The original published data had selectivity values ($\Delta\Delta G^{\ddagger}$) in kcal mol^{-1}. To maintain consistency in this paper we converted these to kJ mol^{-1} by multiplying by a conversion factor of 4.184 J cal^{-1}. To create a graph representation, we took the SMILES representations of the substrate, pre-catalyst, additives (two in total per reaction), and solvents (two in total per reaction) and generated the molecular graphs

in an identical manner to the RhCAA dataset. The CADA database has a wider diversity of atoms, the atom type, atom degree, and hybridization features and reaction conditions. Therefore we included formal Lewis structure charges and reaction concentrations as additional features. In the original paper, 80% of randomly selected reactions are taken as training set and the remainder used for testing. As no specific indication of which reaction was used for each set is given in the literature,[S5] we took our own set of random reactions (80% of the data) as the 'seen' set and those remaining as a final 'unseen' test set. The training set was exposed to a nested-cross validation approach using 10 folds, and the ensemble of models generated was used to get mean predictions of selectivity of the reactions in the test set. This dataset contains only the absolute values of selectivity, and models the reaction:

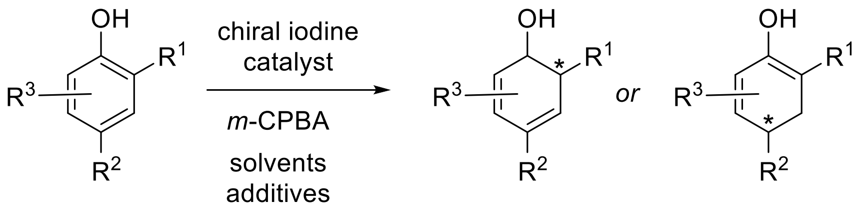

Asymmetric N,S-Acetal Forming Reaction Dataset. The curated dataset was taken from *Li* et al.[S6] This dataset consists of a total of 1075 reactions that combine 5 imines, 5 thiols, and 43 chiral phosphoric acid catalysts. The original dataset had selectivity values ($\Delta\Delta G^{\ddagger}$) in kcal mol^{-1}. To maintain consistency through the paper we converted these values to kJ mol^{-1} by multiplying the values by a conversion factor of 4.184 J cal^{-1}. To create a graph representation, we took the smiles of the thiol, imine, and the catalysts and generated the molecular graphs as we did for the RhCAA dataset. The *N*,*S*-acetal database had a wider diversity of atoms types, so we also included formal Lewis structure charges as an additional feature in our study. Previous publications have modelled selectivity patterns within this dataset (see main paper section HCat-GNet transferability), but all opted to split this dataset into 600 reactions for training, retaining 475 as a test set. As none of these studies stated which 600 points they used as training set, we opted for a random split to generate a 'seen' dataset containing 600 datapoints and the remaining 475 as 'unseen' or final test. We trained the models using a nested cross validation approach using 10 folds from the seen set, and we used the ensemble of models to get a mean prediction of the remaining 475 datapoints. This dataset contains only absolute values of selectivity and models the reaction:

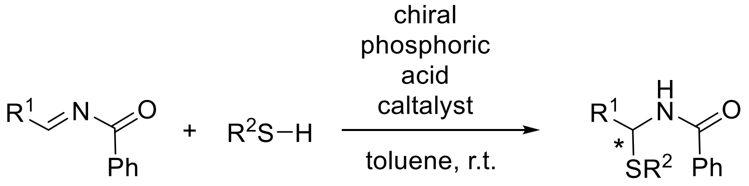

Supplemental references

S1. The 'seen' diene ligand data set is available from: Owen, B.; Wheelhouse, K.; Figueredo, G.; Özcan, E.; Woodward, S. *Results in Chem.* **2022**, *4*, 100379 (doi). These data were curated from ref. S2. All data, including the 'unseen' exemplars from chemical space (nearby but not used in the 'seen' set), are available in the GitHub resources associated with this paper (doi).

S2 Burns, A. R.; Lam, H. W.; Roy, I. D. *Org. React.* **2017**, *93*, 1–415 (doi).

S3 Rit, R.K.; Li, H.; Argent, S. P.; Wheelhouse, K.M.; Woodward, S.; Lam, H.W. *Adv. Synth. Catal.* **2023**, *365*, 1629–1639 (doi).

S4 Li, H. PhD Thesis, A Scalable Synthesis of Chiral Himbert Diene Ligands for Asymmetric Catalysis, University of Nottingham, 2024 (doi).

S5 Gao, B.; Zhang, Y.; Huang, H.; Li, Y.; Xue, X. *CSS Chemistry,* **2024**, *6*, 2515-2528 (doi).

S6 Li, S.W., Xu, L.C., Zhang, C. *et al. Nat Commun*, **2023**, *14*, 3569 (doi).
